# Alternative Polyadenylation Directs Tissue-Specific miRNA Targeting in *Caenorhabditis elegans* Somatic Tissues

**DOI:** 10.1534/genetics.116.196774

**Published:** 2017-03-27

**Authors:** Stephen M. Blazie, Heather C. Geissel, Henry Wilky, Rajan Joshi, Jason Newbern, Marco Mangone

**Affiliations:** * Molecular and Cellular Biology Graduate Program, Arizona State University, Tempe, Arizona 85281; † Virginia G. Piper Center for Personalized Diagnostics, The Biodesign Institute at Arizona State University, Tempe, Arizona 85281; ‡ Barrett Honors College, Arizona State University, Tempe, Arizona 85281; § College of Letters and Sciences, Interdisciplinary Studies, Biological Sciences and Informatics, Arizona State University, Tempe, Arizona 85281

**Keywords:** miRNA, alternative polyadenylation, transcriptome, *C. elegans*

## Abstract

mRNA expression dynamics promote and maintain the identity of somatic tissues in living organisms; however, their impact in post-transcriptional gene regulation in these processes is not fully understood. Here, we applied the PAT-Seq approach to systematically isolate, sequence, and map tissue-specific mRNA from five highly studied *Caenorhabditis elegans* somatic tissues: GABAergic and NMDA neurons, arcade and intestinal valve cells, seam cells, and hypodermal tissues, and studied their mRNA expression dynamics. The integration of these datasets with previously profiled transcriptomes of intestine, pharynx, and body muscle tissues, precisely assigns tissue-specific expression dynamics for 60% of all annotated *C. elegans* protein-coding genes, providing an important resource for the scientific community. The mapping of 15,956 unique high-quality tissue-specific polyA sites in all eight somatic tissues reveals extensive tissue-specific 3′untranslated region (3′UTR) isoform switching through alternative polyadenylation (APA) . Almost all ubiquitously transcribed genes use APA and harbor miRNA targets in their 3′UTRs, which are commonly lost in a tissue-specific manner, suggesting widespread usage of post-transcriptional gene regulation modulated through APA to fine tune tissue-specific protein expression. Within this pool, the human disease gene *C. elegans* orthologs *rack-1* and *tct-1* use APA to switch to shorter 3′UTR isoforms in order to evade miRNA regulation in the body muscle tissue, resulting in increased protein expression needed for proper body muscle function. Our results highlight a major positive regulatory role for APA, allowing genes to counteract miRNA regulation on a tissue-specific basis.

## Abstract (precis)

Alternative polyadenylation (APA) is observed in virtually all metazoans and results in mRNA isoforms with different 3’ends. It is routinely...

MULTICELLULAR organisms rely on sophisticated gene expression programs to confer tissue identity and maintain homeostasis. The mRNA molecule is a dynamic mediator of these programs as it is capable of transferring genetic information into many different isoforms that shape gene expression outputs and precisely direct protein expression. However, in most cases, the dynamics of mRNA expression in the somatic tissues of living organisms that give rise to their specialized functions are still not clear. Thus, mapping tissue-specific transcriptomes of living organisms to identify gene isoforms and their expression levels is key to understanding the how mRNA coordinates development in normal states, and how its expression is disrupted in disease.

The small nematode *Caenorhabditis elegans* is useful for such studies, since it has a complete cell-lineage map (Sulston *et al.* 1983), its development is well studied at the physiological and molecular level (Chalfie *et al.* 1981; Sternberg and Horvitz 1984), it has small, relatively simple tissues, and its transcriptome has been extensively characterized (Gerstein *et al.* 2010; Ramani *et al.* 2011). Its soma is composed of tissue groups commonly found in all metazoans, including muscle, epidermal, epithelial, and neuronal tissues. *C. elegans* has only two muscle tissues (pharynx and body muscle), and two distinct epidermal tissues (hypodermis and seam cells). Its epithelium includes the larger intestine tissue and small connective tissues, such as the arcade and intestinal valve (AIV) cells. *C. elegans* also has a small, yet intricate nervous system composed of 302 cells in adult hermaphrodites, characterized by a range of neural cell types with unique information transmitting abilities.

In recent years, several biochemical approaches were used to isolate, map, and study tissue-specific transcriptomes in *C. elegans*. These studies profiled transcriptome changes spanning from large tissues such as intestine (Pauli *et al.* 2006; McGhee *et al.* 2007; Haenni *et al.* 2012), to smaller tissues composed of just a few cells, such as sensory neurons (Takayama *et al.* 2010). Although such studies have relied on technologies with limited resolution and less reliable quantification (microarrays or tiling arrays), they have highlighted an unexpected complexity of gene regulatory mechanisms used by cells to maintain their tissue identity and to perform their biological roles.

Thus, the application of contemporary sequencing technologies to map and study tissue-specific transcriptomes may greatly expand our understanding of the regulatory mechanisms that establish and maintain cellular identity.

In addition to gene expression levels, small regulatory molecules such as microRNAs (miRNAs) can act as switches for complex developmental regulatory pathways (Lee *et al.* 1993; Reinhart *et al.* 2000; Yoo and Greenwald 2005; Cochella and Hobert 2012; Ebert and Sharp 2012). MiRNAs are short noncoding RNAs that guide the interaction between the RNA-induced silencing complex and target mRNAs by complementary base pairing (“seed”), primarily within the 3′untranslated region (3′UTR) of mRNAs, and typically hold the mRNA targets in translational repression (Bartel 2009). 3′UTRs are the portion of mature mRNAs located between the STOP codon and the poly(A)-tail, and play important roles in the regulation of gene expression (Bartel 2009). MiRNAs and their 3′UTR targets are frequently conserved, and play a variety of roles in regulating fundamental biological processes across metazoans. Recently, several groups have produced tissue-specific localization data for many miRNAs in mouse, rat, and human tissues (Eisenberg *et al.* 2007; Landgraf *et al.* 2007), and in cancer (Jima *et al.* 2010). These results unequivocally show that there are indeed distinct functional miRNA populations in tissues that are, in principle, capable of reshaping transcriptomes and contributing to cell identity acquisition and maintenance. Unfortunately, the tissue-specific gene regulatory networks targeted by miRNAs in living organisms are mostly not known.

3′UTR expression is also dynamically regulated due to a mechanism called alternative polyadenylation (APA), that enables expression of multiple 3′UTR isoforms for the same gene. APA is widespread among eukaryotes (Mangone *et al.* 2010; Jan *et al.* 2011; Shepard *et al.* 2011; Sherstnev *et al.* 2012; Gupta *et al.* 2014), but the mechanisms that direct APA in tissues of living organisms remain poorly understood. APA is achieved through the usage of different polyadenylation signal (PAS) elements distributed within 3′UTRs. PAS elements are hexameric sequences. The canonical PAS (AAUAAA) is the most abundant (∼39%), although permutations of this element are common, and predominant in the short 3′UTR isoforms produced by APA (Mangone *et al.* 2010).

Our group has recently developed a method, called PAT-Seq, that precisely characterizes gene expression signatures in *C. elegans* tissues (Blazie *et al.* 2015). PAT-Seq is an adaptation of the mRNA-tagging method (Roy *et al.* 2002; Spencer *et al.* 2011) coupled with high-throughput sequencing and mapping, and significantly improves the resolution of tissue-specific transcriptome profiling (Blazie *et al.* 2015). In this method, transgenic *C. elegans* nematodes expressing the 3xFLAG-tagged cytoplasmic poly(A)-binding protein (PABPC) in the tissue of interest are used as a bait to crosslink and immunoprecipitate tissue-specific mRNAs. Using PAT-Seq, we have recently profiled the *C. elegans* intestine, pharynx, and body muscle transcriptomes, and produced high quality tissue-specific data that validates, improves and expands previously published datasets (Blazie *et al.* 2015). Importantly, the unique cDNA library preparation methodology used by PAT-Seq limits mispriming events and allowed us to map thousands of high quality tissue-specific polyA sites.

Our recent study of the intestine and muscle tissue transcriptomes revealed the widespread usage of APA in these tissues, including an unexpected abundance of tissue-specific 3′UTR isoform expression that has perhaps a functional role in either promoting or maintaining tissue identity (Blazie *et al.* 2015). Notably, genes expressed with intestine or muscle-specific 3′UTRs are significantly enriched with predicted, and experimentally validated, miRNA targets.

Together, this suggests that crosstalk between APA and miRNA-induced post-transcriptional gene regulation may have a functional role in either promoting or maintaining tissue identity (Blazie *et al.* 2015). While intriguing, the small number of profiled tissues limits any broad conclusions on the nature of this mechanism, and, as such, the interplay between miRNAs and APA in the context of an entire organism is still not clear.

Here, we apply the PAT-Seq approach to isolate and sequence mRNA from five additional *C. elegans* somatic tissues (hypodermis, seam cells, AIV cells, NMDA, and GABA neuronal cells). To allow a direct comparison with our newest datasets, we have also remapped our former PAT-Seq derived muscle and intestine transcriptomes (Blazie *et al.* 2015) to the latest *C. elegans* genome annotation (WS250), gaining an additional 1111 protein-coding genes from these datasets. Our study now follows the tissue-specific dynamics of ∼60% of all experimentally validated *C. elegans* protein-coding genes among eight of their major somatic tissues. Mapping PolyA-sites in these additional tissues confirms widespread tissue-specific APA. We find that, on average, the 3′UTRs of ubiquitously transcribed genes are longer and more enriched with predicted miRNA targets than tissue-restricted genes, suggesting that APA plays a major role in allowing these genes to achieve tissue-specific dosing of their expression. Consistent with this observation, ubiquitously transcribed genes lose ∼37% of predicted miRNA targets to APA events among all tissues. Finally, we provide mechanistic evidence that two human disease gene orthologs, *rack-1* and *tct-1*, use APA in the *C. elegans* body muscle to escape post-transcriptional repression mediated by the ubiquitously expressed *miR-50* miRNA. Together, this data supports a positive regulatory role for APA in modulating targeting of ubiquitously expressed miRNAs, to achieve tissue-specific gene expression at the post-transcriptional level.

## Materials and Methods

### Plasmids and molecular cloning

Molecular cloning of the PolyA-Pull and *∆pab-1*-pull plasmids has been described previously (Blazie *et al.* 2015), and these plasmids were used in this work with no modifications. The tissue-specific promoters used in this study were selected as up to 2 kb of genomic sequence located between the start codon of the target gene and the stop codon of the next closest gene. The primers were designed using the University of Santa Cruz (UCSC) Genome Browser software, with 5-prime Gateway-compatible recombination (Invitrogen) elements for cloning into pDONR P4-P1R entry plasmid (Supplemental Material, Table S4). The DNA promoter elements were amplified using PCR from N2 genomic DNA, and cloned into Gateway pDONR P4-P1R entry plasmids. We used Multisite recombination reactions (LR Clonase II plus, Invitrogen) to combine the tissue-specific promoters with the PolyA-Pull and the *unc-54* 3′UTR into the destination plasmid pCFJ150, which contains the *unc-119* rescue cassette.

The pDONR ROG plasmid was prepared joining the mCherry sequence, a transplicable region between *gpd-2* and *gpd-3*, and the GFP sequence in the pDONR221 vector backbone. The restriction sites used were introduced into pDonr221 using the Stratagene QuikChange Site-Directed Mutagenesis Kit following the manufacturer’s guidelines (Stratagene, La Jolla, CA). All primers used in this study are shown in Table S4. To prepare the pDONR 221 APAreg_1 entry plasmid, we amplified the PEST sequence from pAF207 kindly gifted by Frand *et al.* (2005), using a forward primer containing *Age*I restriction sites, and a reverse primer containing *Kpn*I sites (Table S4). We added *Age*I and *Kpn*I restriction sites downstream of GFP in the pDONR ROG plasmid using the nROGinsAgeIKpnI primers (Table S4), and used them to ligate the amplified PEST sequence downstream of, and in frame with, GFP in the pDONR 221 ROG entry plasmid using NEB Quick ligase (NEB, Ipswich, MA). We observed slightly stronger GFP expression with the pDONR 221 APAreg_1 vector, and used it in experiments with the *tct-1* 3′UTR.

The pDONR 221 APAreg_2 entry plasmid contains the *rpl-10* CDS sequence upstream of, and in frame with, the mCherry and the GFP ORFs, to increase the vector’s nuclear localization. We first added an *Eco*RI restriction site to pDONR 221 ROG upstream of the mCherry using the Stratagene QuikChange Site-Directed Mutagenesis Kit (Stratagene) using the mCherry_ins_*Eco*RI primers and a *Cla*I restriction site downstream of GFP using the GFP_insClaI primers (Table S4). The *rpl-10* sequence was amplified from N2 *C. elegans* genomic DNA using a forward primer containing a *Spe*I restriction site and a reverse primer containing a *Eco*RI restriction site. The amplicon was then ligated upstream of, and in frame with, the mCherry sequence in pDONR ROG using NEB Quick ligase (NEB). To ligate *rpl-10* upstream of, and in frame with, GFP, the *rpl-10* sequence was amplified from N2 *C. elegans* genomic DNA using a forward primer containing a *Sac*II restriction site and a reverse primer containing a *Cla*I restriction site (Table S4). The amplicons were then ligated into pDONR 221 ROG upstream of, and in frame with, GFP coding sequences using NEB Quick ligase (NEB) and named the resulting plasmid pDONR 221 nROG. To add the PEST degron tag sequence downstream of GFP, we amplified its sequence from the pBabe-puro-miR-10b (AddGene plasmid #25043) vector using the PEST primers containing *Age*I and *Kpn*I restriction sites (Table S4). We added *Age*I and *Kpn*I restriction sites downstream of GFP in the pDONR nROG plasmid using the nROGinsAgeIKpnI primers (Table S4), and used them to ligate the PEST sequence downstream of, and in frame with, GFP using NEB Quick ligase (NEB). To prepare the pAPAreg expression vectors, we joined the *myo-3* promoter, each Gateway cassette, and the 3′UTR of interest (see *Preparation of 3′UTR entry plasmids* below) into the Gateway compatible destination plasmid pCFJ150 using Multisite recombination reactions (LR clonase plus II, Invitrogen).

### Preparation of 3′UTR entry plasmids

To clone the *rack-1* 3′UTR sequence into the Gateway pDONR P2R-P3 entry plasmid, the *rack-1* 3′UTR was amplified from N2 genomic DNA using the *rack-1*_3′UTR forward and *rack-1*_3′UTR reverse primers flanked by 5′ Gateway recombination sequences (Table S4). The *tct-1* 3′UTR sequence was amplified from N2 genomic DNA using the *tct-1*_3′UTR_fwd and *tct-1*_3′UTR_rev primers flanked by 5′ Gateway recombination sequences (Table S4). To remove the proximal polyadenylation signal element (PAS1) from the 3′UTRs of *rack-1* and *tct-1* needed to force expression of their long 3′UTR isoforms, we used the Stratagene QuikChange Site-Directed Mutagenesis Kit (Stratagene) using the primers *rack-1*_delPAS_fwd and *rack-1*_delPAS_rev (for *rack-1*), or *tct-1*_delPAS_fwd and *tct-1*_delPAS_rev (for *tct-1*) (Table S4). These primers replaced the PAS elements in these 3′UTRs with a *Bgl*II restriction site to maintain the length of the long 3′UTR isoform. To further remove the predicted *miR-50* miRNA seeds from *rack-1* and *tct-1* 3′UTRs, we used the Stratagene QuikChange Site-Directed Mutagenesis Kit (Stratagene) using the primers *rack-1*_delmiR50_fwd and *rack-1*_delmiR50_rev (for *rack-1*) and *tct-1*_delmiR50_fwd and *tct-1*_delmiR50_rev (for *tct-1*) (Table S4). We used the same strategy to delete the predicted *miR-85* miRNA seed from the *rack-1* 3′UTR using the primers *rack-1*_delmiR85_fwd and *rack-1*_delmiR85_rev (Table S4). The resulting clones from each experiment were verified using Sanger sequencing.

### Preparation of nematode strains

The EG4322 [*ttTi5605* II; *unc-119(ed9)* III] *C. elegans* strain, which we used to prepare PolyA-Pull expressing transgenic lines, was maintained at 16° on NGM plates seeded with *Escherichia coli* HB101 bacteria prior to microinjection. Extrachromosomal array transmitting *C. elegans* lines (one each tissue, total five lines) were prepared by microinjecting pCFJ150 tissue-specific Promoter::GFP::*pab-1*::*unc-45* 3′UTR (25 ng/μl) along with the markers pCFJ90 (1 ng/μl), pGH8 (10 ng/μl), pCFJ104 (5 ng/μl) as carrier DNA constructs to promote formation of complex extrachromosomal arrays into the background *C. elegans* strain EG4322 [*ttTi5605* II; *unc-119(ed9)* III], which were kindly provided by Priscilla Van Wynsberghe (Colgate University, Hamilton, NY). Microinjection was carried out as described previously (Mello *et al.* 1991) using a Leica DMI3000B microscope. A complete list of strains prepared in this study, and their full genotypes with WormBase-approved laboratory nomenclature, is provided in Table S5.

### RNA immunoprecipitation

Mixed stage cultures of each transgenic *C. elegans* line were grown in liquid culture at 20° as described (Stoeckius *et al.* 2009). Transgenic animals harvested from liquid culture were crosslinked in formaldehyde, and flash frozen as previously described (Blazie *et al.* 2015). *C. elegans* lysates were prepared as follows: each pellet was thawed, centrifuged for 30 sec at 10,000 rpm, and resuspended in lysis buffer (150 mM NaCl, 25 mM HEPES pH 7.5, and 0.2 mM dithiothreitol). Samples were then sonicated on ice five times (10 sec pulses, 18 W RMS output), with 50 sec pauses between pulses. Lysates were centrifuged at 16,000 × *g* for 15 min at 4°. The supernatant from each lysate was used for immunoprecipitation of mRNA, which was carried out as previously described (Blazie *et al.* 2015). Each sample was quantified using the Nanodrop Instrument (Thermo-Fisher Scientific), and subsequently used in RT-PCR reactions and cDNA library preparation for sequencing.

### RT-PCR reactions

For each tissue-specific RNA sample, 100 ng RNA was reverse transcribed with an NVdT_(23)_ primer (Table S4) using Superscript Reverse Transcriptase III (Thermo-Fisher Scientific), according to the manufacturer’s protocol; 1 μl of the resulting cDNA was used as a template for each second DNA strain reaction, along with 1 μM of gene-specific primer (Table S4) and *Taq* DNA Polymerase (NEB) to drive the reactions.

### cDNA library preparation and sequencing

The 10 cDNA libraries were prepared using 50 ng of total RNAs for each tissue. IntegenX’s (Pleasanton, CA) automated Apollo 324 robotic preparation system was used to reverse transcribe RNA into cDNA, and for cDNA library preparation, as previously described (Blazie *et al.* 2015). The cDNA synthesis was performed using a SPIA (Single Primer Isothermal Amplification) kit (IntegenX and NuGEN, San Carlos, CA) (Kurn *et al.* 2005). The cDNA Shearing was performed on a Covaris S220 system (Covaris, Woburn, MA). After the cDNA was sheared to ∼300 bp fragments, we used the Nanodrop instrument to quantify the cDNAs and calculate the appropriate amount of cDNA necessary for library construction. Tissue-specific barcodes were then added to each cDNA library. The resulting 10 tissue-specific libraries were then pooled and sequenced using the HiSeq platform (Illumina, San Diego, CA) with a 1 × 50 bp HiSeq run.

### Raw reads mapping

Reads were demultiplexed by their unique tissue-specific barcodes and converted individually to FASTQ files by the CASAVA software (Illumina). Unique datasets were mapped to the *C. elegans* gene model WS250 using the Bowtie software (Langmead *et al.* 2009) run with default parameters. A summary of the results produced by this approach is shown in Tables S1 and S2 in File S1. Mapped reads were further converted into a bam format, and sorted using SAMtools software run with generic parameters (Li *et al.* 2009). For the intestine and muscle tissues, we downloaded the original raw reads from the NCBI Sequence Read Archive (http://trace.ncbi.nlm.nih.gov/Traces/sra/), SRP Study Accession # SRP044802, and remapped the original raw “sorted” BAM files used previously (Blazie *et al.* 2015), using Bowtie (Langmead *et al.* 2009) and Cufflinks (Trapnell *et al.* 2012) with standard parameters, to the WS250 gene model.

### Cufflinks analysis

We used Cufflinks software to calculate expression levels of individual transcripts (Trapnell *et al.* 2012). We used the fragment per kilobase per million base (FPKM) number to assign the gene expression levels. For tissues with extrachromosomal arrays (hypodermis, seam cells, AIV cells, GABAergic neurons, and NMDA neurons), we used a median FPKM value ≥1 as a threshold for defining expressed genes. For the tissues profiled using integrated lines (intestine, pharynx, and body muscle) we used an FPKM value ≥1 assigned by Cufflinks that is normalized between replicates. Genes with an FPKM <1 were ignored in our analysis.

### 3′RACE reactions

For each N2 total RNA or intestine or body muscle-specific RNA sample [from RNA-IP of *ges-1*::PAP and *myo-3*::PAP transgenic animals, (Blazie *et al.* 2015)], 100 ng RNA was reverse transcribed with a NVdT_(23)_ primer containing a 5′anchor sequence (Mikl and Cowan 2014) (Table S4) using Superscript Reverse Transcriptase III (Thermo-Fisher Scientific), according to the manufacturer’s protocol; 1 μl of the resulting cDNA was used as a template for each PCR reaction, along with 1 μM of gene-specific forward primer for *rack-1* or *tct-1*, the anchor reverse primer (Table S4), and *Taq* DNA Polymerase (NEB) to drive the reactions.

### Nematode imaging and fluorescence quantification

The fluorescence produced by extrachromosomal array in *C. elegans* strains carrying the pAPAreg transgene was detected using a Leica DMI3000B microscope. Images were captured using a Leica DFC345FX mounted camera with Gain = 1×, Gamma = 0.5, and 1 sec exposure. GFP/mCherry fluorescence ratios were quantified using the integrated density (ID) function of ImageJ software (Hartig 2013) using the formula [ID_GFPt_ − [(ID_GFPb_/Area_b_) × Area_t_)] − ID_GFP_N2_]/[(ID_mCherry_t_ − ((ID_mCherry_b_/Area_b_) × Area_t_) − ID_mCherry_N2_] where: ID_GFPt_ and ID_mCherry_t_ are the integrated density values of each transgenic nematode image obtained from GFP and mCherry channels, respectively. ID_GFPb_ and ID_mCherry_b_ are the integrated density values obtained from a small selection of the dark area (background) surrounding *C. elegans* animals in each image. Area_b_ is the area of this small background selection, and Area_t_ is the total area of the entire image. ID_GFP_N2_ and ID_mCherry_N2_ are the average integrated density values obtained from nonfluorescent N2 animals (*n* = 15) in the GFP or mCherry channels, respectively.

### RNA interference assays

Each RNAi clone was obtained from the Julie Ahringer library (Kamath *et al.* 2003) and the RNAi by feeding procedure was performed as described (Timmons *et al.* 2001). Briefly, each RNAi clone was grown in LB overnight at 37°, at 1000 rpm. Each clone was seeded on small NGM medium plates supplemented with 1 mM IPTG, and activated overnight at room temperature. To observe embryonic lethality and uncoordinated phenotypes, 10 L4 stage *myo-3*::PolyApull expressing transgenic *C. elegans* strains (Blazie *et al.* 2015) were plated onto the seeded plates and incubated at 20° for 24 hr. Adult *C. elegans* animals were then singled onto new plates seeded with the same RNAi clones. After incubation at 20° for 12 hr, the adults were removed, and larval offspring continued to incubate for 24 hr before scoring their phenotypes.

### Poly(A)-cluster building and mapping

Poly(A)-clusters were built using custom Perl scripts. We extracted FASTQ sequence reads containing at least 23 adenosines at their 3′ ends, removed the A’s and mapped the remaining sequence (≥10 nts) to the WS250 *C. elegans* genome annotation using Bowtie (Langmead *et al.* 2009). For each aligned read, we selected 5 nts upstream and downstream of the sequence region surrounding the 3′ end of each mapped read, and built Poly(A)-clusters from overlapping 3′end sequence fragments using BedMerge (Quinlan and Hall 2010). We ignored Poly(A)-clusters mapping to regions containing ≥65% adenosines within 20 nt of the end of each cluster. Each Poly(A)-cluster was then bioinformatically attached to the closest WS250 gene on the same strand within no >100 nt downstream of the furthest WS250 defined 3′UTR end. We merged Poly(A)-clusters mapping within ≤5 nt across all the datasets. We ignored Poly(A)-clusters having <5% of the total reads for all clusters in a given 3′UTR.

### Comparison with other GABAergic and hypodermis datasets

For the GABAergic dataset, we used a list of 242 genes ranked by expression levels present in the original Cinar *et al.* (2005) dataset obtained from the supplementary materials section of the publisher, and compared with our GABAergic neuron dataset. For the hypodermis dataset, we compared the list of 1234 hypodermis genes published by Spencer *et al.* (2011) with our hypodermis specific dataset.

### miRNA target prediction analysis

We downloaded *C. elegans* miRNA target prediction data from the PicTar (Lall *et al.* 2006) and miRanda (Betel *et al.* 2008) databases, and obtained the miRNA name and target coordinates for each mapped 3′UTR of each gene. To study the enrichment of predicted miRNA family targets between tissue transcriptomes, miRNA targets were grouped into their families (Alvarez-Saavedra and Horvitz 2010) using custom VBA scripts in Microsoft Excel. For this analysis, we focused on the general enrichment of *C. elegans* miRNA families, which have the same “seed” sequence used to target the mRNA (Alvarez-Saavedra and Horvitz 2010), and ranked the enrichment of their targets in genes expressed within each tissue transcriptome.

### Data availability

Raw reads were submitted to the NCBI Sequence Read Archive (http://trace.ncbi.nlm.nih.gov/Traces/sra/), under the SRP Study Accession no. SRP075984. The results of our analyses are available in Excel format as Table S2 in File S1 and Table S3, and in our 3′UTR-centric website http://tomato.biodesign.asu.edu/cgi-bin/UTRome/utrome.cgi.

## Results

### Deep sequencing of five additional *C. elegans* somatic tissue transcriptomes

**Figure 1:**
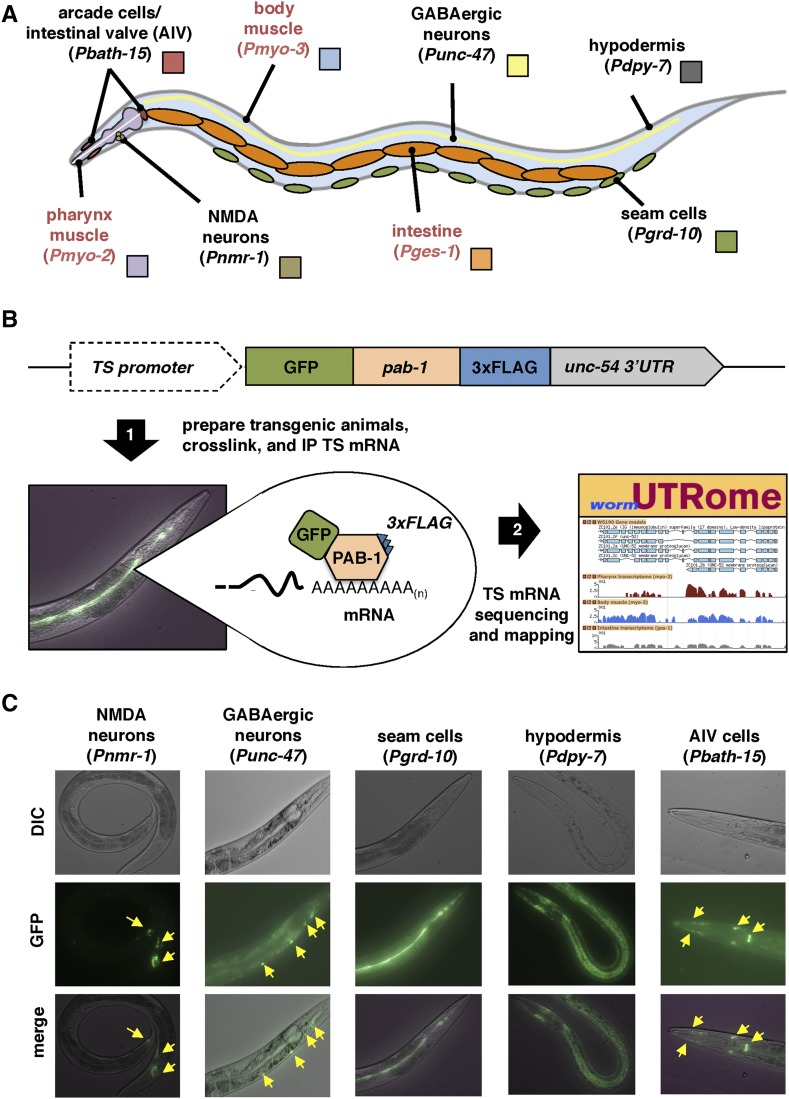
PAT-Seq to study the transcriptomes of AIV cells, NMDA-type neurons, GABAergic neurons, seam cells, and hypodermis. (A) Diagram with the anatomical location of tissues we have profiled in this study. Red labels mark tissues that our group has previously profiled (Blazie *et al.* 2015), and that have been remapped in this study and used in the analysis. (B) Overview of the PAT-Seq approach. The tissue-specific expression of the Poly(A)-Pull cassette containing GFP fused to 3×FLAG-tagged *pab-1* is achieved through usage of selected tissue-specific promoters. Transgenic *C. elegans* lines that express this construct are then prepared and grown in liquid culture, followed by crosslinking, lysis and immunoprecipitation of 3×FLAG-tagged PAB-1 complexes. The tissue-specific mRNA extracted from the IP is then used to prepare cDNA libraries for next generation sequencing and transcriptome mapping, which is stored in the UTRome.org database (Mangone *et al.* 2008). (C) Examples of fluorescent images of transgenic lines expressing the Poly(A)-Pull cassette in the tissue profiled in this study. Yellow arrows mark small cells expressing the construct.

We have applied our PAT-Seq approach to isolate, sequence, and map tissue-specific mRNA transcripts from five *C. elegans* somatic tissues: hypodermis, seam cells, GABAergic neurons, NMDA neurons, and the epithelial tissue surrounding the pharynx that includes the arcade and intestinal valve (AIV) cells (Figure 1A). Together, these tissues cover much of the *C. elegans* anatomy, allowing us to perform a more comprehensive sampling of tissue-specific expression changes and study their APA dynamics. To further enrich our analysis, we have also incorporated updated datasets from our previously mapped intestine, pharynx, and body muscle transcriptomes (Blazie *et al.* 2015) (Figure S1, A and B in File S1).

In the PAT-Seq approach, the *C. elegans* cytoplasmic polyA-binding protein (*pab-1*) is fused with GFP and a 3xFLAG epitope (PolyA-Pull), and expressed in the tissue of interest using tissue-specific promoters (Figure 1B). The mRNA bound to the PolyA-Pull construct from transgenic *C. elegans* strains is then crosslinked and immunoprecipitated using anti-FLAG antibodies, and then deep sequenced (Figure 1B).

In order to ensure efficient immunoprecipitation of mRNA from significantly smaller tissues with very few cells, we adjusted our PAT-Seq approach to make it more sensitive. In our original protocol, we used the Mos-1 single copy insertion technology to prepare stable transgenic *C. elegans* lines with a genome integrated PolyA-Pull cassette (Frokjaer-Jensen *et al.* 2008; Blazie *et al.* 2015). While this method guarantees homogenous expression of the transgene, the low expression levels gained from single copy insertions may not allow PolyA-Pull expression at levels sufficient for RNA pull-down in tissues composed of just a few cells. We therefore prepared transgenic *C. elegans* lines expressing PolyA-Pull from multicopy extrachromosomal arrays, which robustly express the transgenes in somatic tissues (Stinchcomb *et al.* 1985). We have also implemented a sonication step to improve the *C. elegans* lysis following crosslinking (see *Materials and Methods*) (Grosshans *et al.* 2005).

We have prepared five transgenic *C. elegans* lines using tissue-specific promoters to drive expression of the PolyA-Pull construct in hypodermis (*dpy-7* promoter), seam cells (*grd-10* promoter), GABAergic neurons (*unc-47* promoter), NMDA-expressing neurons (*nmr-1* promoter), and the AIV cells (*bath-15* promoter) (Figure 1C). After crosslinking and immunoprecipitation, we used an RT-PCR approach to confirm the enrichment of tissue-specific mRNA (Figure S1C in File S1). As expected, the *dpy-7* and *grd-10* transcripts were selectively enriched in hypodermis and seam cell mRNA preparations, respectively, while the pharynx muscle gene *myo-2* and the neuronal genes *unc-47* and *nmr-1* were absent from these samples (Figure S1C in File S1). These data indicate that our updated PAT-Seq protocol enriches for tissue-specific transcripts and limits mRNA background from other tissues.

We then prepared cDNA libraries from two biological replicates of each tissue (total 10 samples). As in our previous application of PAT-Seq (Blazie *et al.* 2015), we used the Single Primer Isothermal Amplification (SPIA) cDNA library preparation methodology, to improve transcriptome mapping quality, as it produces cDNAs using small amounts of mRNA and limits mispriming artifacts (Kurn *et al.* 2005; Blazie *et al.* 2015) (see *Materials and Methods*). We pooled and barcoded the 10 tissue-specific cDNA libraries, and sequenced them using the Illumina Hi-Seq Instrument.

**Figure 2:**
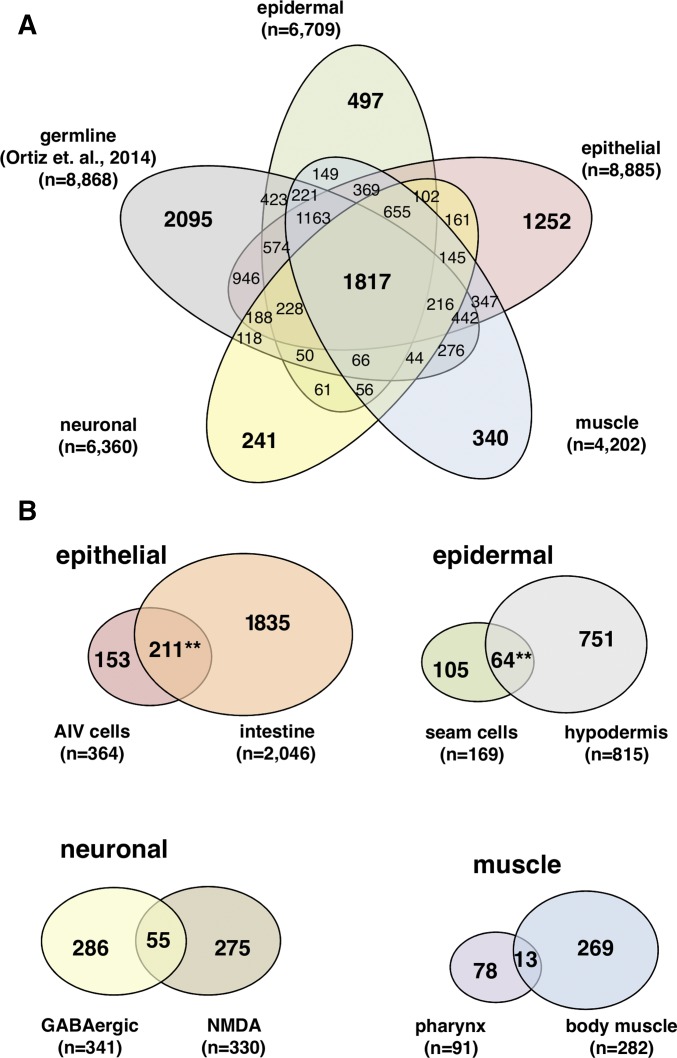
Distribution of gene expression among eight somatic tissues profiled with PAT-Seq. (A) Venn diagram displaying the portions of genes expressed between four tissue groups we have profiled in this study and the germline transcriptome from Ortiz *et al.* (2014). Tissues from our study were grouped by muscle (pharynx and body muscle), neuronal (GABAergic and NMDA-type neurons), epithelial (intestine and AIV cells), and epidermal (hypodermis and seam cells) groups. (B) Venn diagram of the epithelial-unique (Top left), epidermal-unique (Top right), neuronal-unique (Bottom left), and muscle-unique genes (Bottom right) identified by PAT-Seq. Epithelial tissue (AIV cells and intestine) and the epidermal tissue (seam cells and hypodermis) transcriptomes were highly similar (**P < 0.01, hypergeometric distribution).

We mapped our five tissue-specific transcriptomes to the latest *C. elegans* WS250 genome annotation release (Howe *et al.* 2016) (see *Materials and Methods*). Since our intestine and muscle-derived sequencing reads were previously mapped onto the older *C. elegans* WS190 release (Blazie *et al.* 2015), we also remapped these datasets to WS250 to allow direct comparison between all transcriptomes we have profiled so far (red text in Figure S1B and Table S1 in File S1). Our overall strategy successfully mapped the majority of raw reads produced from our tissue-specific transcriptome sequencing (Table S1 in File S1). Genes and their expression levels from both biological replicates for each tissue are well correlated at the high-end where our gene expression cut-off was made (Figure S2 and Table S2 in File S1, and *Materials and Methods*). Principle component analysis further supports the correlation between tissue replicates (Figure S2B in File S1). In total, our analysis has identified 11,481 protein-coding genes (∼60%), and assigned them to each of the five tissues profiled (Figure 2A).

### Extensive overlap in genes expressed between the intestine and the AIV cells

Several *C. elegans* epithelial tissues link tissue borders and specify anatomical boundaries, such as the AIV cells, which anchor the anterior pharynx to the hypodermis and attach the posterior pharynx to the intestine (Mango 2007; Rasmussen *et al.* 2013). These cells play an important role in pharyngeal extension during early development of the digestive tract (Portereiko and Mango 2001). Other epithelial cells have more specialized roles, such as the intestine, which supports digestion, innate immunity, and dauer formation (McGhee 2007). The majority of the genes identified by PAT-Seq are expressed in these two epithelial tissues (8885 genes, 77% of the total genes detected in this study) (Figure 2A). When compared with the six other somatic tissue transcriptomes profiled in our study (excluding the germline), a large pool of 2199 genes is uniquely expressed in the epithelial tissues (Figure 2B). As previously reported by our group and others, these tissues are generally enriched with metabolic enzymes, proteins supporting innate immunity, and the GATA transcription factors (Pauli *et al.* 2006; McGhee *et al.* 2007; Haenni *et al.* 2012; Blazie *et al.* 2015) (Table S2 in File S1). We found 3601 genes to be expressed in the AIV cells (Figure 1A and Table S3). This dataset is enriched in genes involved in embryonic development (*eef-1A.2*, *lev-11*, *icd-1*, and others) and lifespan (*dao-6*, *ril-1*, *pghm-1*, and others). Within this group, we also identified 153 genes that are uniquely expressed in this tissue (Figure 2B) including several transcription factors with poorly understood roles that may direct the development or function of these cells (Table S3).

Interestingly, a large portion of 211 genes is uniquely coexpressed between this tissue and the intestine (Figure 2B). The unexpected similarity between these two tissues may reflect the common roles of intestinal valve cells and the intestinal tract posterior to it. Three of the seven most abundantly expressed genes in this category (*sptf-2*, *nhr-106*, and *elk-2*) are transcription factors that we speculate may play a role in gut formation.

Notably, a surprisingly large pool (64 members) of F-box domain containing genes are expressed uniquely in the intestine. These genes are known to participate in epithelial-to-mesenchymal transitions, are commonly misregulated in cancers (Diaz and de Herreros 2016), and are potentially involved in the aging process (Ayyadevara *et al.* 2009) and protein homeostasis in *C. elegans* (Horn *et al.* 2014; McCue *et al.* 2015).

### Genes expressed in the hypodermis and seam cells are associated with a vast array of functional activities

*C. elegans* has two distinct epidermal tissues, hypodermis and seam cells, which play important roles in larval development. The hypodermis is a large epidermal tissue composed of 138 nuclei in the adult hermaphrodite (Chisholm and Hardin 2005). This tissue has multiple functions, including forming the cuticle and basement membranes, directing neuronal placement and axon pathfinding, regulating the development of neighboring cells, removing apoptotic cells, and establishing the body plan; 6033 genes are expressed in this tissue (∼30% of all protein-coding genes in WS250) (Table S3). Importantly, our hypodermis transcriptome overlaps extensively with a published dataset from Spencer *et al.* (2011), who performed mRNA-tagging experiments in this tissue using tiling arrays (Figure S3A in File S1). Consistent with its function in cuticle formation, we have identified 87 collagen genes previously shown to coordinate precise molting events through development (Jackson *et al.* 2014). We also identified many hedgehog-related genes that are also expressed in the hypodermis, and thought to contribute to cuticle formation in *C. elegans* (Hao *et al.* 2006); 920 genes are uniquely expressed in the epidermis (Figure 2B). Many of these genes have been previously described in WormBase (Stein *et al.* 2001), and are known to have roles in molting and embryonic development.

Highlighting the role of the epidermal cells in relaying information from the environment, we found several metabolic regulators of neurotransmitter synthesis or transport with previously described epidermal functions, such as *snf-3* (Peden *et al.* 2013) and *snf-12* (Dierking *et al.* 2011), as well as serpentine receptor genes, which are important chemosensory molecules (McGrath *et al.* 2011).

Importantly, only 751 genes are uniquely expressed in the hypodermal cells (Figure 2B). Aside from the many genes involved in cuticle formation, this pool is enriched in genes involved in molting (F42A8.1, *fkb-5*, *mlt-10*, *mlt-2*, and others), lifespan and growth rate (*old-1*, *osm-1*, *nphp-4*, F56D5.5 and others), embryonic development (C03B8.2, C17E7.4, C46A5.5, *nphp-4* and others), and solute carriers/transporters (K08H10.6, *snf-12*, T11G6.3, *vglu-2*, *vglu-3*, and others). We also detected several transcription factors that are either known to control larval development like *ceh-16* (Cassata *et al.* 2005) and *nhr-23* (Kouns *et al.* 2011) or have putative roles in hypodermal cell fate (Y73F8A.24, *ztf-23*, *atf-8*, *attf-5* and others). Over 31% of these 751 genes (238 genes) have no assigned function.

A more specialized epidermal tissue located laterally along the anterior-posterior axis, called seam cells, coordinate larval transitions through asymmetric divisions that form hypodermal or neural cells depending on their position along the lateral axis (Chisholm and Hardin 2005). Consistent with a role in epidermal activities, the seam cells express 52 collagen genes, several other molting genes, and eight hedgehog-like genes that are known to function in this tissue (Hao *et al.* 2006) (Table S3). In contrast with the hypodermis, the seam cells express a much smaller pool of 105 unique genes (Figure 2B). Two genes in this group—*ceh-10* and *ceh-43*—are homeobox transcription factors primarily known to be involved in neural fate specification (Altun-Gultekin *et al.* 2001; Aspock and Burglin 2001) and may promote neuroblast formation resulting from seam cell asymmetric divisions in development (Joshi *et al.* 2010). Of the genes uniquely expressed in seam cells, 67 have no function described so far and need to be further investigated.

### Only 9% of genes in the GABAergic and NMDA-type neuronal transcriptomes are coexpressed in these tissues

*C. elegans* neurons possess a diverse array of information-transmitting capabilities that rely upon the expression of specific neurotransmitters or neurotransmitter receptors. Twenty-six neurons expressing the neurotransmitter gamma-aminobutyric acid (GABA) are required for locomotion and defecation (McIntire *et al.* 1993; Schuske *et al.* 2004; Jorgensen 2005). The transcriptome of this tissue has been profiled in the past using a microarray approach, which identified over 250 genes (Cinar *et al.* 2005). Our PAT-Seq approach correlates with, and expands, these results (Figure S3B in File S1 and Table S3). Out of the total 4885 genes identified in GABAergic neurons, 286 (∼6%) are uniquely expressed in this tissue, and include potassium channels, GABA regulatory genes (*unc-47*, *snf-11*), and neurotransmitter receptors (*lgc-35*, *lgc-34*, *ggr-3*, *lev-1*, *acr-15*, and *gar-3*) (Figure 2B). Eighty-six genes in this list currently lack WormBase gene function annotations, indicating their roles are unknown. Interestingly, the putative transcription factors, *nfya-1*, *madf-1*, *unc-86*, and *lin-29* were uniquely and highly expressed in GABAergic neurons, suggesting a possible role in the transcriptional specification or maintenance of GABAergic neuron identity.

Six interneurons (AVA, AVD, AVE, RIM, AVG, PVC) express the N-methyl-d-aspartate (NMDA) receptor subunit, *nmr-1*, and support locomotion and memory (Brockie *et al.* 2001; Kano *et al.* 2008). In the NMDA neurons, we detected many G-protein signaling components (*dmsr-3*, *dmsr-6*, *rab-37*, and others) and potassium channels (*twk-16*, *twk-17*, and *twk-39*) consistent with a neuronal phenotype. Interestingly, we mapped eight genes belonging to the nematode-specific peptide families that do not have functions described for this tissue. This pool of genes also includes several transcription factors, such as the poorly characterized T-box transcription factor *tbx-34*. We also detected many *C. elegans* orthologs of human neuronal disease genes. One of these genes is *ceh-6*, a homolog of human POU3F4 transcription factor commonly mutated in conductive deafness (de Kok *et al.* 1995) (OMIM: 304400). Another, *mbtr-1*, contains human malignant brain tumor repeats (OMIM: 608802) that, when mutated in *Drosophila*, leads to malignant transformation of the larval brain (Janic *et al.* 2010).

The neuronal pool is smaller than the epidermal and epithelial one (6360 genes), with 616 genes exclusively expressed in either the GABAergic or NMDA neuronal cells (Figure 2A). Surprisingly, only 55 genes are uniquely coexpressed in both neuronal subtypes (Figure 2B). This lack of similarity in their gene expression profiles may reflect the distinct functional differences between these tissues despite their common neuronal identity. Among the top hits in this list of neuron-specific shared genes are insulin family members (*ins-1* and *ins-17*), previously shown to be expressed in neurons through development (Pierce *et al.* 2001), and G-protein signaling components (*rgs-6*, M04G7.3, *srsx-25*, and others). Notably, one of the transcription factors detected in this list is *mab-9*, which is a T-box transcription factor gene known to be enriched in motor neurons and important for axon guidance (Pocock *et al.* 2008; Jafari *et al.* 2011). We have also detected other transcription factors, such as *hlh-1* and *nhr-67* that may be broadly important for specifying neural identity. Importantly, 13 genes (∼24%) in this list do not have a known function. These candidates may be important regulators of broader aspects of neuronal identity or function.

### The refined *C. elegans* muscle transcriptome reveals 360 uniquely expressed genes

The pharynx and body muscle, which support foraging and locomotion, are the only two muscle tissues in *C. elegans* (Chen *et al.* 1992; Mango 2007). Our remapping of the muscle transcriptomes from our past study detected 4202 genes in muscle tissues, and only 360 (8.5%) of these genes are uniquely expressed in muscle cells (Figure 2A and Table S3). In addition to myosin and other known muscle genes, such as lectins, that are known to play a role in locomotion (Link *et al.* 1992), we identified genes that regulate neurotransmitter responses and postsynaptic activity in neuromuscular junctions (Seifert *et al.* 2006). While the majority of muscle genes are exclusive to the body muscle tissue, 78 are uniquely expressed in the pharynx (Figure 2B). The body muscle-specific gene pool contains 269 genes (Figure 2B). Similar to what we previously reported, we detected a small overlap of only 13 genes expressed in common between pharynx and body muscle (Figure 2B). Interestingly, three of these shared genes (*zip-8*, *bed-1*, and *klu-2*) are putative transcription factors that we speculate may be important for conferring basic muscle identity. The unique shared expression of these transcription factors in both tissues suggests that they merely regulate the additional 10 genes coexpressed in these tissues. However, it is possible that other factors including their post-translational regulation or cofactor requirements, may enable them to *trans*-activate different genes in each tissue. Additional experiments are needed to further address their roles in these tissues.

### APA is widespread in *C. elegans* somatic tissues

**Figure 3:**
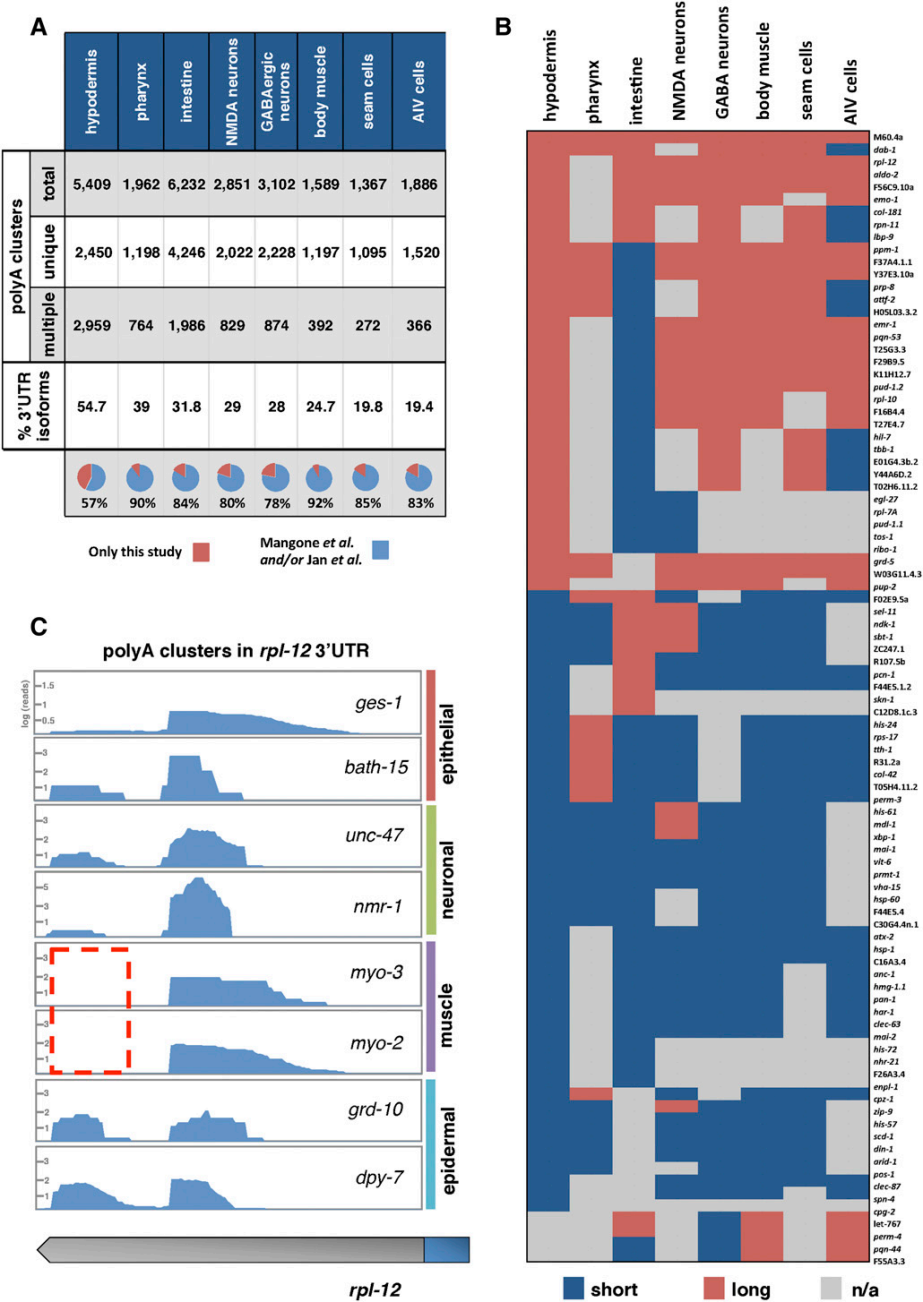
PolyA Cluster mapping. (A) We have bioinformatically assembled ∼25,000 high-quality polyA clusters distributed in eight somatic *C. elegans* tissues from our RNA-seq data. These clusters map the 3′ends of protein coding genes. Genes in these tissues use APA at a ∼31% rate; >83% of these mapped polyA clusters map 3′UTR isoforms previously detected by Mangone *et al.* (2010) and Jan *et al.* (2011) (B) Tissue-specific 3′UTR isoform preferences in 91 protein coding genes detected with only two 3′UTR isoforms in this study. Most of the genes in this analysis use tissue-specific APA. Instances where a 3′UTR was not mapped in a tissue are indicated in gray (n/a). (C) Example of polyA clusters prepared for the gene *rpl-12*. The distal 3′UTR isoform is absent in both muscle tissues (dotted red box).

We recently showed that 3′UTR isoforms generated through APA are frequently expressed in a tissue-specific manner in *C. elegans* intestine, pharynx, and body muscle tissues (Blazie *et al.* 2015). In order to expand and refine our past results, we decided to map the 3′ends of these five additional transcriptomes and study their dynamics across each somatic tissue. For this analysis, we have employed an improved poly(A)-cluster building strategy (see *Materials and Methods*), which allowed us to precisely map 15,956 unique, high-quality poly(A) clusters across all eight somatic tissues (Figure 3, A–C). Our results show that APA is widespread in *C. elegans* somatic tissues, with an average of 31% of genes that express alternate 3′UTR isoforms in a tissue-specific manner. This result mirrors our previous findings in three tissues (Blazie *et al.* 2015) (Figure 3A), with nearly 81.5% of these polyA sites corroborated by previous studies (*C. elegans* 3′UTRome) (Mangone *et al.* 2010; Jan *et al.* 2011) (Figure 3A). 3′UTR isoform switching between tissues is also highly dynamic (Figure 3B). An example of cluster structure and 3′UTR isoform switching for the ubiquitously expressed gene, *rpl-12*, is shown in Figure 3C.

The general distribution of 3′UTR length is strikingly similar among each tissue (Figure S4A in File S1). However, the intestine transcriptome is enriched with a larger pool of shorter 3′UTRs that are <200 nt long (Figure S4A in File S1). The median 3′UTR length is similar between common tissue types, where the two muscle tissues have the longest median 3′UTR length (>200 nts), followed by the two neuronal tissues (>190 nts) (Figure S4B in File S1). 3′UTRs expressed in the intestine and hypodermis possess the shortest 3′UTR length (Figure S4B in File S1).

### Commonly transcribed genes are expressed with longer 3′UTRs that are frequently subject to APA and post-transcriptional regulation by miRNAs

**Figure 4:**
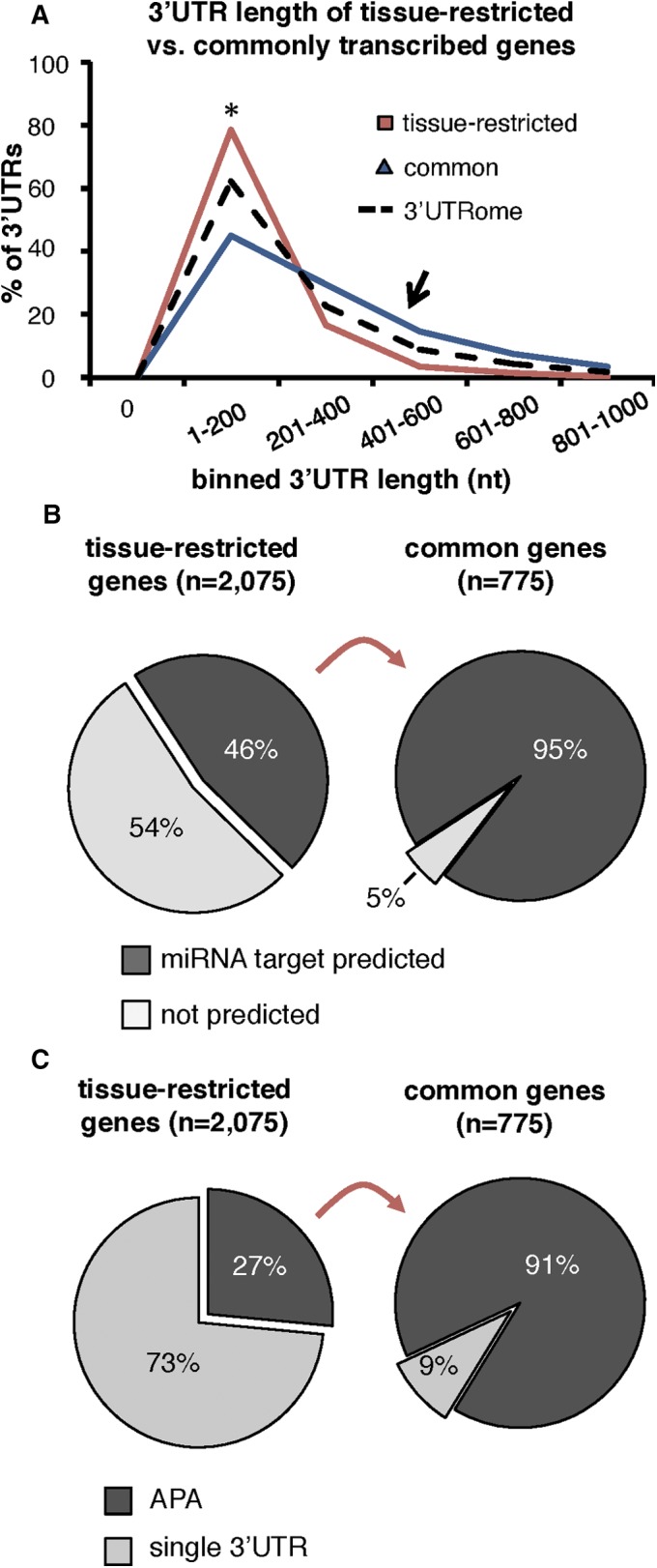
Commonly transcribed genes are enriched in APA and miRNA targets. (A) Histogram comparing the length distribution of 3′UTRs of tissue-restricted (red) and commonly transcribed genes (blue) with the *C. elegans* 3′UTRome (dotted black). Commonly transcribed genes have longer 3′UTRs, on average. (B) Portion of commonly transcribed or tissue-restricted genes with at least one PicTar or mirANDA predicted miRNA target in their 3′UTRs. Most commonly transcribed genes have at least one predicted miRNA target. (C) Pie charts displaying the proportion of tissue-restricted genes (left chart) or commonly transcribed genes (right chart) with >1 3′UTR isoform (APA). Nearly all commonly transcribed genes are prepared with at least two 3′UTR isoforms.

Our improved poly(A) mapping strategy identified poly(A)-clusters for 2075 tissue-restricted genes and 775 commonly transcribed genes within our eight tissues. When we compared the length of the 3′UTRs from each of these groups with data from the *C. elegans* 3′UTRome, we found that 3′UTRs of tissue-restricted genes are on average shorter in length, while 3′UTRs of commonly transcribed genes are instead longer (Figure 4A). We reasoned that the generally long 3′UTRs in commonly transcribed genes might also possess more miRNA targets than the 3′UTRs of tissue-restricted genes. Indeed, nearly all (95%) of commonly transcribed genes have at least one PicTar (Lall *et al.* 2006) or miRANDA (Betel *et al.* 2008) predicted miRNA target (Figure 4B), suggesting that these genes have a greater capacity for post-transcriptional gene regulation by miRNAs. Strikingly, only 46% of tissue-restricted genes are predicted targets of miRNAs (Figure 4B).

Surprisingly, we found that 91% of commonly transcribed genes are expressed with multiple 3′UTR isoforms and use APA between the eight tissues profiled in this study (Figure 4C). In contrast, a much smaller portion (27%) of tissue-restricted genes use APA (Figure 4C), and these genes use noncanonical PAS elements at a rate of 60% (Figure S4C in File S1), which is consistent with the previously published *C. elegans* 3′UTRome average (Mangone *et al.* 2010). Commonly transcribed genes instead use noncanonical PAS elements for 74% of cleavage events (Figure S4C in File S1), reflecting the higher rate of APA in these genes, since in *C. elegans* these PAS elements are frequently used to induce APA (Mangone *et al.* 2010); 37% of miRNA targets are lost in these 3′UTRs because of tissue-specific APA (Figure S4D in File S1).

Since several miRNA families are known to be abundant in the *C. elegans* soma (Alvarez-Saavedra and Horvitz 2010), we hypothesized that APA may be used by genes to counteract their widespread negative regulatory role. The relative abundance of miRNA family targets among each tissue is largely similar, where targets of the miRNA families *miR-2*, *mir-58.1*, and *let-7* dominate the top three most enriched miRNAs in almost every tissue (Figure S5 in File S1, left). Very few miRNA families (ex. *miR-1* and *miR-51*) change widely in enrichment rank between tissues (Figure S5 in File S1, left). In contrast, we observed dramatic tissue-specific differences in miRNA target loss in genes that use APA. In particular, predicted targets of miRNA families *mir-72*, *mir-232*, and *mir-87*, which have a relatively low abundance in each transcriptome (Figure S5 in File S1, left), are instead frequently lost between tissues (Figure S5 in File S1, right). Our data suggest that APA allows a significant rearrangement of miRNA targeting events in each tissue transcriptome, presumably to coordinate tissue-specific modulation of gene expression.

### Commonly transcribed genes rack-1 and tct-1 escape post-transcriptional regulation by miRNAs in the body muscle

Our study reveals that APA is more abundant in genes that are transcribed in multiple tissues, and enhances the usage of noncanonical PAS elements in these genes. In addition, we found that APA causes the loss of miRNA targets in shorter 3′UTR isoforms. We hypothesized that the abundant usage of APA among commonly transcribed genes may provide them with a platform to selectively escape post-transcriptional gene regulation by miRNAs in a tissue-specific context.

**Figure 5:**
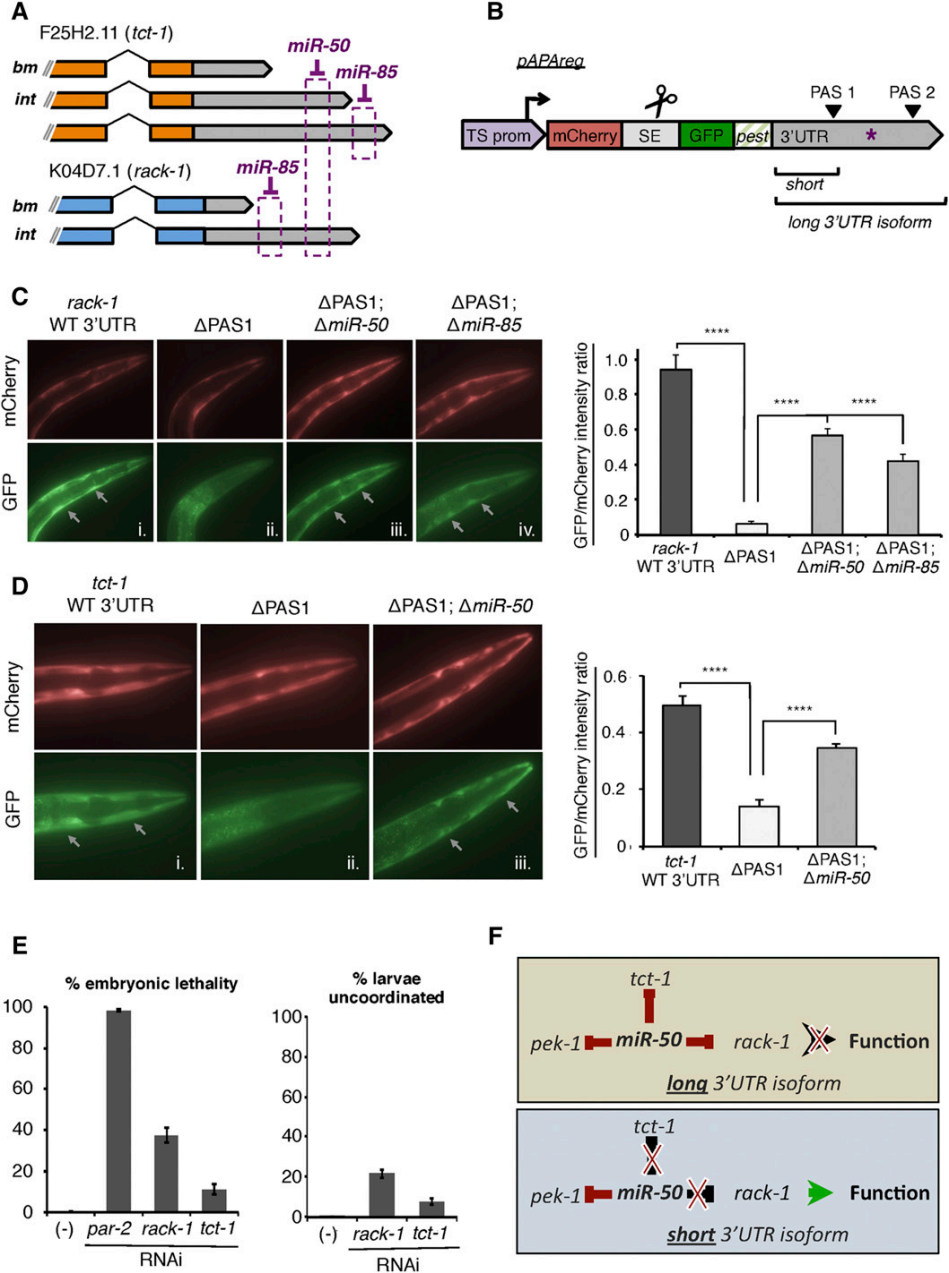
Tissue-specific APA events allow *rack-1* and *tct-1* to escape miRNA mediated gene repression in body muscle. (A) Illustration (not to scale) of all 3′UTR isoforms identified in our study for *tct-1* (orange) and *rack-1* (blue) with the location of PicTar predicted miRNAs targets. We detected their short 3′UTR isoforms in the body muscle (bm) and the long isoforms exclusively in the intestine (int). (B) pAPAreg: A dual-color reporting system to study post-transcriptional gene regulation *in vivo*. The system uses the Gateway multisite technology. When expressed in *C. elegans* using tissue-specific promoters, the operon cassette (SE) is cleaved, and both fluorochromes are expressed in the same molar ratio in a given tissue. After the *trans*-splicing of the spliceable element (SE), the mCherry fluorochrome is translated independently of GFP-PEST, which is instead subject to post-transcriptional repression via miRNAs that target (purple asterisk) in the 3′UTR placed downstream of it. Deletion of PAS1 allows expression of the long 3′UTR isoform containing the miRNA target in the body muscle where it is not normally expressed. (C) *rack-1* escapes *miR-50* and *miR-85* regulation in the body muscle through APA. Left: Representative mCherry and GFP fluorescent images of transgenic lines expressing pAPAreg in the body muscle using the *myo-3* promoter and the *rack-1* 3′UTR with (i) wild-type sequence (WT), (ii) deleted PAS1 (ΔPAS1), (iii) deleted PAS1 and *miR-50* target (ΔPAS1;Δ*miR-50*), or (iv) deleted PAS1 and *miR-85* target (ΔPAS1;Δ*miR-85*). Right: Quantification of GFP fluorescence intensity relative to mCherry for each *rack-1* transgenic *C. elegans* line pictured in left panel using Image-J software (*n* = 34, ****P < 0.0001, paired *T*-test). (D) *tct-1* escapes *miR-50* regulation in the body muscle through APA. Left: Representative mCherry and GFP fluorescent images of transgenic *C. elegans* lines expressing pAPAreg in the body muscle using the *myo-3* promoter and the *tct-1* 3′UTR with (i) wild-type sequence (wt), (ii) deleted PAS1 (ΔPAS1), or (iii) deleted PAS1 and *miR-50* target (ΔPAS1;Δ*miR-50*). Right: Quantification of GFP fluorescence intensity relative to mCherry for each *tct-1* transgenic *C. elegans* line pictured in left panel using Image-J software (*n* = 27, ****P < 0.0001, paired *T*-test). (E) RNAi mediated knockdown of *rack-1* and *tct-1* results in partial embryonic lethality, (*n* = 10). *C. elegans* animals fed *rack-1* or *tct-1* RNAi exhibit uncoordinated locomotion. Shown are the results from larval *C. elegans* animals that bypassed embryonic lethality (*n* = 10). (F) Model depicting the manner in which APA allows *rack-1* and *tct-1* to escape regulation by the *miR-50* miRNA in a tissue-specific manner.

In order to test this hypothesis, we studied the consequence of APA events involving two ubiquitously transcribed genes, *rack-1* and *tct-1*, which are representatives of a pool of genes identified in this study that exhibit tissue-specific 3′UTR isoform expression (Figure 5A). *rack-1* is the *C. elegans* ortholog of the widely conserved receptor for activated C-kinase, and is expressed in the body muscle with a short 3′UTR isoform spanning 41 nts from the STOP codon to the poly(A)-tail (Figure S6A in File S1). We mapped an alternate APA event for this gene in the intestine that produces a longer 3′UTR isoform spanning 78 nts (Figure S6A in File S1).

*tct-1* is the *C. elegans* ortholog of the translationally controlled tumor protein gene. This gene is localized in the endoplasmic reticulum and known to be involved in growth, locomotion, and embryo and larval development (WormBase) (Meyvis *et al.* 2009). We mapped a shorter 3′UTR isoform for *tct-1* in the body muscle and a longer 3′UTR isoform expressed exclusively in the intestine (Figure S6B in File S1). We initially confirmed the differential tissue expressions of *rack-1* and *tct-1* 3′UTR isoforms using 3′RACE (Figure S6, A and B in File S1).

We used PicTar miRNA target predictions to search for potential targets in *rack-1* and *tct-1* 3′UTRs (Lall *et al.* 2006; Mangone *et al.* 2010). This analysis revealed predicted targets for *miR-50*, a ubiquitously expressed miRNA, in both genes (Figure 5A). *miR-50* is predicted to target the most distal portion of the long 3′UTR isoform of *rack-1*, nearest the distal PAS element that is expressed in intestine (Figure 5A). This target is not present in the short 3′UTR isoform expressed in the body muscle tissue. The same miRNA is also predicted to target a long 3′UTR isoform of *tct-1* that is expressed in the intestine, and unable to target the short 3′UTR isoform expressed in the body muscle tissue (Figure 5A). Notably, PicTar software also predicts a miRNA target for *miR-85* in the longest 3′UTR isoform of *tct-1* and *rack-1* (Figure 5A). PicTar also predicts a *miR-50* target in another gene, *pek-1*, that is expressed in the same tissues with one 3′UTR isoform, suggesting it is part of the same network targeted by *miR-50*.

In our model, *tct-1* and *rack-1* escape *miR-50* and *miR-85* regulation in the body muscle through APA-induced 3′UTR shortening. To investigate this hypothesis, we developed a unique vector-based sensor tool (pAPAreg) that can sense post-transcriptional gene regulation by miRNAs *in vivo* in transgenic *C. elegans* strains (Figure 5B). pAPAreg contains two fluorochromes, the mCherry and the Green Fluorescent Protein (GFP), separated by a *trans*-spliceable element (SE), derived from the well-characterized sequence region between *trans*-spliced genes *gpd-1* and *gpd-2* in the *mai-1* operon (Zorio *et al.* 1994) (Figure 5B). We placed the body muscle-specific promoter, from the gene *myo-3*, upstream of this construct to allow transcription of a polycistronic pre-mRNA followed by SL2 *trans*-splicing between the mCherry and GFP fluorochromes. The mCherry fluorochrome serves as a transcriptional reporter since it is spliced away from GFP, and it is not subject to conditions between experiments. In contrast, the GFP fluorochrome is downregulated if a miRNA target (purple asterisk in Figure 5B) is present and used in the long 3′UTR isoform, and therefore acts as a translation reporter (Figure 5B). Importantly, this tool includes a degron tag (Frand *et al.* 2005) placed downstream of the GFP fluorochrome, to limit its protein stability and allow detection of subtle repressive events mediated by miRNAs (Figure 5B).

We have used this sensor tool to detect post-transcriptional gene regulation due to body muscle-specific APA events in *rack-1* and *tct-1* (Figure 5, C and D). Body muscle expression of wild-type (short) *rack-1* 3′UTR resulted in unabated expression of GFP throughout *C. elegans* developmental stages (Figure 5Ci). This isoform does not contain the *miR-50* binding site. We then forced the expression of the long *rack-1* 3′UTR isoform by deleting the proximal PAS element (PAS1) used to express the short 3′UTR isoform, and observed strong GFP repression in the body muscle (Figure 5Cii). We then expressed the long 3′UTR isoform containing a deletion of the 5 nt that pair with the *miR-50* seed required for targeting, and observed a significant rescue of GFP expression (Figure 5Ciii). We also observed a significant rescue in GFP expression after deleting the predicted *miR-85* seed from the distal portion of the long 3′UTR isoform (Figure 5Civ). Taken together, these results provide evidence that *rack-1* escapes *miR-50*- and *miR-85*-induced repression in body muscle using APA.

We then tested the effect of forcing the long 3′UTR isoform of *tct-1* in the body muscle (Figure 5D). Similar to our results with *rack-1*, the usage of wild-type *tct-1* allowed GFP expression through all developmental stages, with no obvious changes in levels (Figure 5Di). We observed a significant decrease in GFP expression when forcing the long *tct-1* 3′UTR isoform after deleting its proximal PAS element (PAS1) (Figure 5Dii). GFP levels were significantly rescued when we expressed the long 3′UTR isoform of *tct-1* after deleting the predicted *miR-50* seed in the distal portion of the 3′UTR (Figure 5Diii).

Together, these results provide strong evidence that *rack-1* and *tct-1* use body muscle-specific APA events to evade miRNA regulation through *miR-50*, presumably to dose their expression to levels required for their role in the body muscle.

We hypothesized that *rack-1* and *tct-1* use a short 3′UTR to escape miRNA regulation in body muscle because high protein levels for these genes are needed for proper viability. We tested whether lowering their protein levels in this tissue would interfere with proper *C. elegans* muscle development. Knockdown of *rack-1* using RNAi resulted in ∼40% embryonic lethality (Figure 5E). Similarly, knockdown of *tct-1* resulted in embryonic lethality at a rate of ∼10% (Figure 5E). These results suggest that variation of protein levels in these two genes interferes with the overall viability and potentially embryo hatching, since viable body muscles in needed for larvae to break the egg shell in this process. Importantly, we also observed variable uncoordinated phenotypes in several young *C. elegans* larvae that were able to bypass embryonic lethality, further suggesting an important role for RACK-1 and TCT-1 proteins in locomotion (Figure 5E). These results mirror those obtained in similar knockout studies (Meyvis *et al.* 2009). Together, our results support a model where *rack-1* and *tct-1* use APA to counteract miRNAs in the body muscle tissue and allow their expression to support locomotion (Figure 5F).

### Abundant APA within and between tissues induces predicted miRNA target loss

Our study suggests that commonly transcribed genes, like *rack-1* and *tct-1*, use APA to escape miRNA regulation on a tissue-specific basis to fine-tune their expression (Figure 5F). Since our study showed that genes transcribed in a wide range of tissues are particularly prone to APA usage, and are also enriched in predicted miRNA targets (Figure 4, B and C), we reasoned that APA might act as a positive regulator of their gene expression by decreasing the amount of miRNA targets, thus allowing them to elude miRNA regulation. We downloaded miRNA target prediction data from PicTar (Lall *et al.* 2006) and miRANDA (Betel *et al.* 2008) databases and over imposed them to the tissue-specific 3′UTRs mapped from our eight tissue transcriptomes (see *Materials and Methods*). Of all the tissue-specific miRNA targets, 37% are lost because of APA events (Figure S4D in File S1). This suggests that, within this pool, not only *rack-1* and *tct-1*, but also a large portion of genes in *C. elegans* somatic tissues could potentially use APA to evade miRNA regulation and dose their expression.

## Discussion

In this study, we have profiled, analyzed, and now distribute to the Community, the transcriptome and 3′UTRome of *C. elegans* hypodermis, seam cells, GABAergic and NMDA neurons, AIV cells, intestine, pharynx, and body muscle tissues. We have followed the expression dynamics of ∼60% (almost 12,000 genes) of all *C. elegans* protein-coding genes, and produced high-quality tissue-specific datasets to map APA across multiple cell types. We used a very stringent gene-mapping filter (see *Materials and Methods*), which, in turn, may have excluded many low expressed genes. As expected, we did not detect ∼2000 germline-specific genes known to be expressed in the *C. elegans* gonads (Ortiz *et al.* 2014; Stoeckius *et al.* 2014) (Figure 2A). Within our datasets, we noticed an unexpected low number of genes that are shared between tissues with similar identity (Figure 2B). *C. elegans* somatic tissues are highly specialized, and perhaps require only a few common genes to establish their group identity, while instead significantly more genes are required for their specialized functions. Our remapping of the intestine and muscle genes to the WS250 release significantly expanded the number of genes detected in these tissues, gaining an additional 1111 genes not previously detected in our past study (Blazie *et al.* 2015). The addition of five somatic tissue transcriptomes allowed us to further refine these gene pools, assigning only 78 pharynx-specific genes, 269 genes in the body muscle, and 1643 genes specific to the larger intestine tissue.

We have previously demonstrated PAT-Seq sensitivity and specificity in large tissues (Blazie *et al.* 2015). Our further refined PAT-Seq analysis can now successfully profile small tissues that range from just a few cells, such as the AIV cells and NMDA-type neurons. Importantly, the results from two independent biological replicates in each of these tissues, correlate well, further suggesting that results produced by PAT-Seq are consistent (Figure S2 in File S1). Using seam cells as an example, we used an RT-PCR approach to show that our RNA pull-down enriches for tissue-specific transcripts, with little background from surrounding tissues (Figure S1 in File S1). Our results are also consistent with other past studies, which also used RNA pull-downs from small *C. elegans* tissues such as neurons (Cinar *et al.* 2005; Kunitomo *et al.* 2005; Spencer *et al.* 2011) (Figure S3 in File S1). Sequencing the mRNA from these tissues enabled us to detect significantly more genes with increased sensitivity compared to past studies. This is reflected by our sequencing results from GABAergic neurons, which correlate with the top expressed genes from Cinar *et al.* (2005) and significantly expand the number of genes detected in this tissue from just over 200 genes to almost 5000 genes (Figure S3B in File S1). Although PAB-1 based approaches may prefer binding transcripts with longer polyA-tails leading to bias in the resulting transcriptomes, our results from each neuron tissue correlate very well with a recent dataset from Kaletsky *et al.* (2016), who instead used neuron isolation procedures. Our results highlight PAT-Seq as a sensitive and specific method for profiling small tissue transcriptomes in *C. elegans*.

APA has been reported in a variety of tissues and developmental contexts (Tian and Manley 2013). However, the biological function of APA has been described in only a handful of cases and remains largely unexplored, especially in living organisms. For example, in murine quiescent satellite cells (QSCs), the differentiation-inducing transcription factor Pax3 is maintained at low levels due to the broad expression of *miR-166*, a miRNA that targets the distal portion of its 3′UTR. In a subset of QSCs, APA of Pax3 enables *miR-166* target exclusion, allowing it to counteract miRNA repression and dose its expression to levels sufficient to induce muscle cell differentiation (Boutet *et al.* 2012).

We have shown that the ubiquitously expressed *C. elegans* genes *rack-1* and *tct-1* use APA to escape miRNA regulation by *miR-50*. While tissue level expression data for this miRNA is not available, its coding locus resides in an intron of the *C. elegans* gene *tln-1*, which is known to be expressed in body muscle cells (Moulder *et al.* 1996) and suggests *miR-50* is also expressed in body muscle. Of note, we also detected *tln-1* abundantly expressed in every tissue we have profiled with the exception of pharynx and seam cells, indicating that *miR-50* is widely expressed in the *C. elegans* soma. Unfortunately, *miR-50* loss-of-function mutants are not viable (Miska *et al.* 2007), preventing further validation of this network using a complementary analysis with *C. elegans* strains lacking *miR-50*. The *miR-85* deletion strain MT12999 *miR-85(n4117)* is indeed available. When injected with the *rack-1* long 3′UTR isoform containing a deletion of the *miR-85* binding site, this strain was able to rescue the lack of GFP fluorescence in N2 animals injected with *rack-1* long 3′UTR isoform alone (Figure S7 in File S1).

Although we have followed only two cases, the widespread nature of APA in *C. elegans* highlighted by this study, and its correlation with miRNA enrichment, suggests this mechanism is operating on a much wider level that what is currently appreciated.

Previous work has shown that 3′UTR isoform expression achieved though APA is rarely observed in absolute levels. Instead, genes may modulate ratios of multiple 3′UTR isoforms in the same tissue with varying abundance (Lianoglou *et al.* 2013; Tian and Manley 2013). Importantly, a large population of genes detected in this study use APA in this manner (Figure 3C). In these cases, APA may still buffer gene expression by managing the relative portions of transcripts that contain regulatory targets, thereby fine-tuning their expression. In this view, genes that favor shorter 3′UTR isoforms induce a net increase in gene expression compared with other tissues where longer 3′UTR isoforms are more abundant.

Additionally, it is possible that long 3′UTR isoforms containing miRNA targets are also expressed in tissues where we have mapped only the short isoform, and that these transcripts are subsequently degraded due to miRNA activity. Our approach likely does not detect such transcripts since they are only transiently expressed. While we cannot make assertions that the presence of a given miRNA can induce APA *per se*, the presence of the short 3′UTR isoform in the same tissue indicates that these genes are in fact able to escape miRNA regulation to appropriately buffer their expression.

These results are also supported by a recent survey of APA events in a panel of human cancer cell lines and tissues of various origin (Lianoglou *et al.* 2013). While diverse in terms of samples and datasets, this work suggests that APA seems to be frequently used as a mode of post-transcriptional regulation among commonly transcribed genes (Lianoglou *et al.* 2013). This study also reported an interesting correlation where genes commonly transcribed among tissues may use APA to counteract the repression mediated by ubiquitously expressed miRNAs.

Here, we finally validate this observation in an intact organism and in disease-free states. Our results from eight *C. elegans* somatic tissues show that commonly expressed transcripts indeed possess generally longer 3′UTRs with more predicted miRNA targets than tissue-restricted genes.

We note that target prediction algorithms may over-predict targets in genes having long 3′UTR sequences based on random chance. To minimize the interference of such false positives, we used miRNA target prediction algorithms that utilize stringent criteria, including conservation of the query sequence to make predictions (Lall *et al.* 2006; Betel *et al.* 2008). While we cannot rule out that some targets are inevitably predicted in a given sequence by chance, such filters substantially limit such artifacts from adversely influencing our results.

Our examination of the relative enrichment of predicted miRNA targets that are lost in genes with APA include broadly expressed miRNAs such as *lin-4* and *let-7*, suggesting this may also be the case in *C. elegans*. It is not clear, however, whether the expression pattern for many of the other miRNAs is tissue-ubiquitous or tissue-restricted. Further experiments that address their specific expression patterns will shed light on this model. Unfortunately, tissue-specific proteomic data in *C. elegans* is limited, and we are unable to correlate our tissue-specific expression data, and APA events to tissue-specific protein changes, which is how miRNA activity is executed.

Another interesting hypothesis is that mRNA 3′end formation is linked to mRNA splicing, such that CDS isoforms expressed through alternative splicing are also expressed with specific 3′UTR isoforms due to APA. While intriguing, the widely used 3′end sequencing techniques developed so far such as PolyA-capture (Mangone *et al.* 2010), 3P-Seq (Jan *et al.* 2011), 3′READS+ (Zheng *et al.* 2016), 3′seq (Lianoglou *et al.* 2013), and others, are generally biased toward the 3′end of transcripts, making it impossible to simultaneously map ORF and APA sequence information. Future approaches that address this challenge will shed more light on the possibility that 3′UTR and splice isoform expression are congruent.

In this study, we have used PAT-Seq to profile the transcriptome and APAome of five *C. elegans* tissues (hypodermis, seam cells, NMDA neurons, GABAergic neurons, and AIV cells), and to study the impact of APA on tissue-specific miRNA activities. We have also incorporated in our analysis previously published datasets from our laboratory, of the intestine, pharynx, and body muscle datasets for an indepth analysis of a total of eight somatic *C. elegans* tissues (Blazie *et al.* 2015). This work now provides the community with the first systematic and comprehensive deep sequenced tissue-specific transcriptome and APAome resource ever performed in a living organism, significantly improving and expanding the *C. elegans* tissue-specific tiling array data published by the modENCODE Consortium in 2011 (Spencer *et al.* 2011).

Here, we show that APA is highly dynamic among somatic *C. elegans* tissues. Nearly all ubiquitously expressed transcripts use APA and harbor miRNA targets in their 3′UTRs, and these miRNA targets are frequently lost in a tissue-specific manner due to 3′end shortening.

Using a novel set of tools to allow the systematic study of APA *in vivo*, we show that two ubiquitously transcribed human disease ortholog genes use APA to evade miRNA regulation in the body muscle *in vivo*, dosing their expression to levels required for body muscle function. Since these two genes were among a large set of ∼700 commonly transcribed genes identified in our screen, we conclude that APA has a greater regulatory role than suspected, by allowing genes to counteract miRNA repression on a tissue-specific basis. This study is of particular significance because it not only demonstrates the biological role that APA plays in gene regulation, but also provides *proof-of-principle* of the utility of this resource for the larger community.

We have uploaded and release our tissue-specific transcriptomes and APA data through our 3′UTRome database (http://tomato.biodesign.asu.edu/cgi-bin/UTRome/utrome.cgi) (Mangone *et al.* 2008, 2010; Blazie *et al.* 2015). The 3′UTRome database is a public repository of *C. elegans* 3′UTR annotation, hosted at Arizona State University, which offers a simple and well-integrated interactive user interface to query gene records and 3′UTR isoform data at a tissue-specific level. This database now displays tracks for each tissue transcriptome, including tissue-specific APA, as well as curated 3′UTR data from previously published studies (Mangone *et al.* 2008; Jan *et al.* 2011; Haenni *et al.* 2012).

## Supplementary Material

Supplemental material is available online at www.genetics.org/lookup/suppl/doi:10.1534/genetics.116.196774/-/DC1.
