# Supplementary material for: Alternative Polyadenylation Directs Tissue-Specific miRNA Targeting in Caenorhabditis elegans Somatic Tissues
Source: Genetics. 2017 Mar 27;206(2):757–74. doi: 10.1534/genetics.116.196774 (PMC5499184; doi:10.1534/genetics.116.196774)
Supplement: Supplementary file 1 [file 757FileS1.pdf]

Supplemental Data for:

Alternative polyadenylation directs tissue specific miRNA targeting in *Caenorhabditis elegans* somatic tissues

Stephen M Blazie, Heather C Geissel, Henry Wilky, Rajan Joshi, Jason Newbern and Marco Mangone*

* To whom correspondence should be addressed. Tel: (480) 965-7957; Email: mangone@asu.edu

This PDF includes:

Figures S1-S7

Table S1 - Summary of results from PAT-Seq after deep sequencing.

Table S2 - Summary of sequencing results after mapping genes to WS250

Table of Contents

Figure S1: Poly(A)-Pull validation and comparison with WS250 ………..….3

Figure S2 : PAT-Seq sequencing results………………………………….....4

Figure S3: PAT-Seq comparison with other published mRNA-tagging derived datasets……..6

Figure S4: 3'UTR length in each tissue and PAS usage in tissue-restricted versus commonly transcribed genes……..…7

Figure S5: APA drives loss of distinct miRNA family targets……….….......8

Figure S6: *rack-1* and *tct-1* are expressed with a short 3'UTR isoform in the body muscle and long 3'UTR isoform in the intestine….9

Figure S7: *rack-1 3'UTR* ΔPAS1;Δ*miR-85* construct rescues GFP expression in *miR-85*(n4117)………………..…...........10

Table S1: Summary of results from PAT-Seq after deep sequencing......11

Table S2: Summary of sequencing results after mapping genes to WS250...12

Figure S1

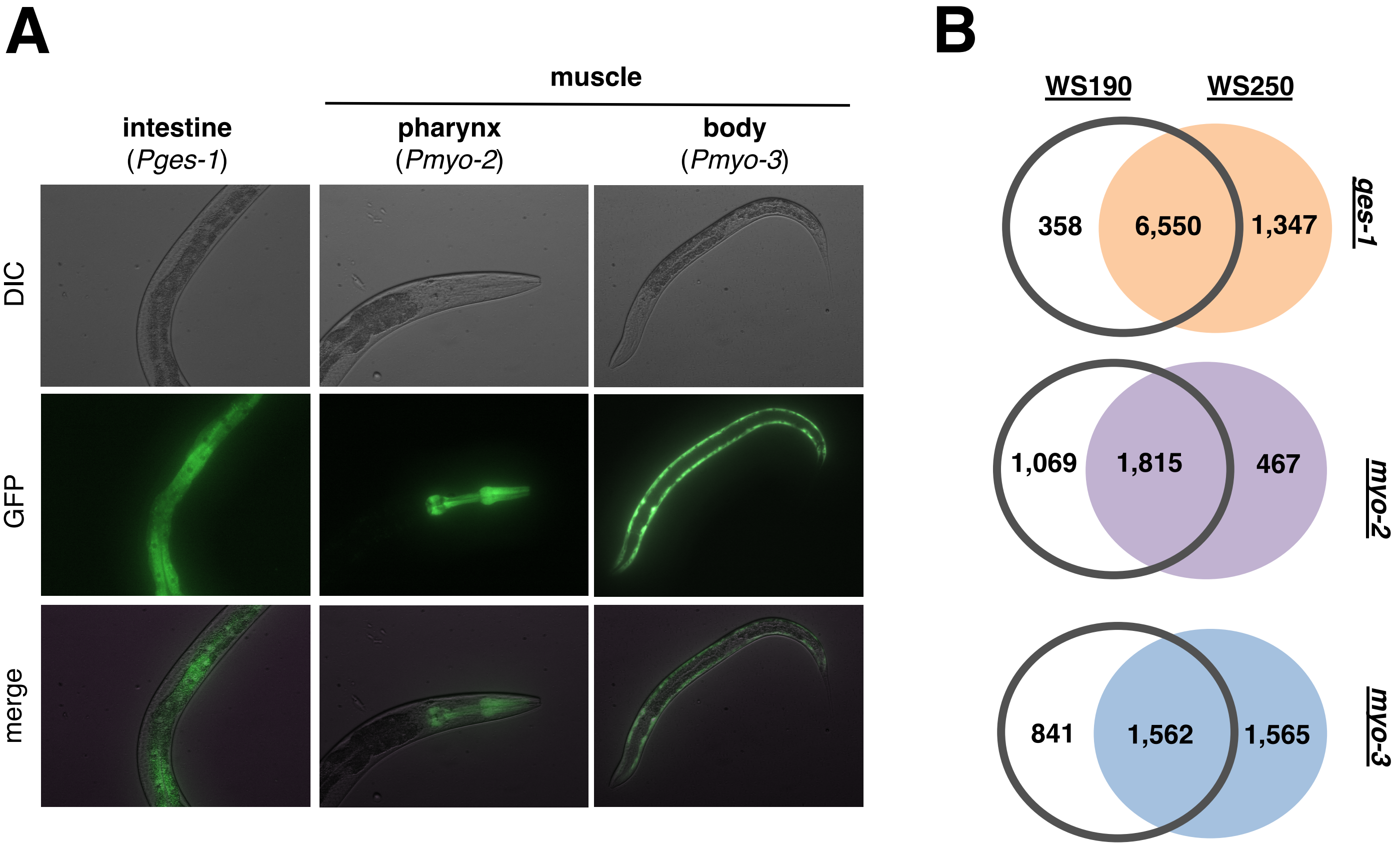

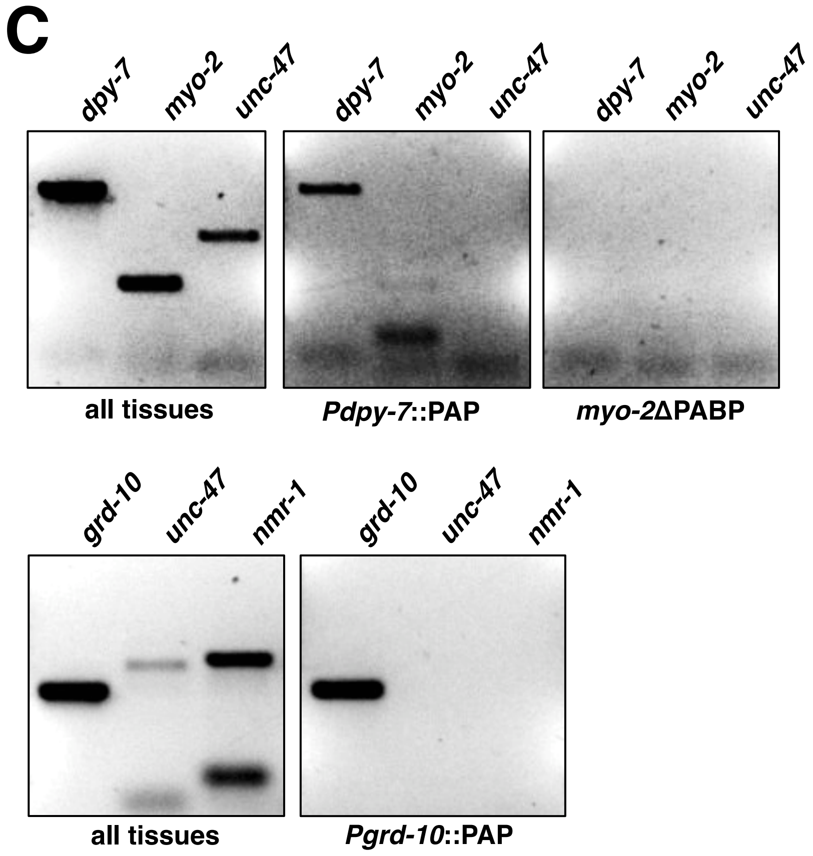

Figure S1:Poly(A)-Pull validation and comparison with WS250. A) Transgenic worms expressing the Poly(A)-Pull construct in intestine, pharynx, and body wall muscles. We previously applied PAT-Seq to study the transcriptomes of these three tissues (Blazie et al., 2015), which are now included in this study. **B)** Genes mapped in intestine and muscles from *Blazie et al. 2015* using WS190 annotations compared to those we have now remapped to WS250. **C)** RT-PCR experiments validating the specificity of mRNA pulldown using Poly(A)-pull expressed in hypodermis (*dpy-7*) and seam cells (*grd-10*). We detected *dpy-7*, *myo-2*, and *unc-47* transcripts in total RNA from all tissues, while *dpy-7* is specifically enriched in mRNA prepared from *dpy-7*::PAP worms. The same transcripts were not detected in mRNA immunoprecipitated using our negative control construct lacking PABPC (*myo-2*ΔPABP). We detected seam cell specific *grd-10*, but not neuronal genes *unc-47* or *nmr-1* from mRNA immunoprecipitated from worms expressing Poly(A)-Pull in the seam cells.

Figure S2

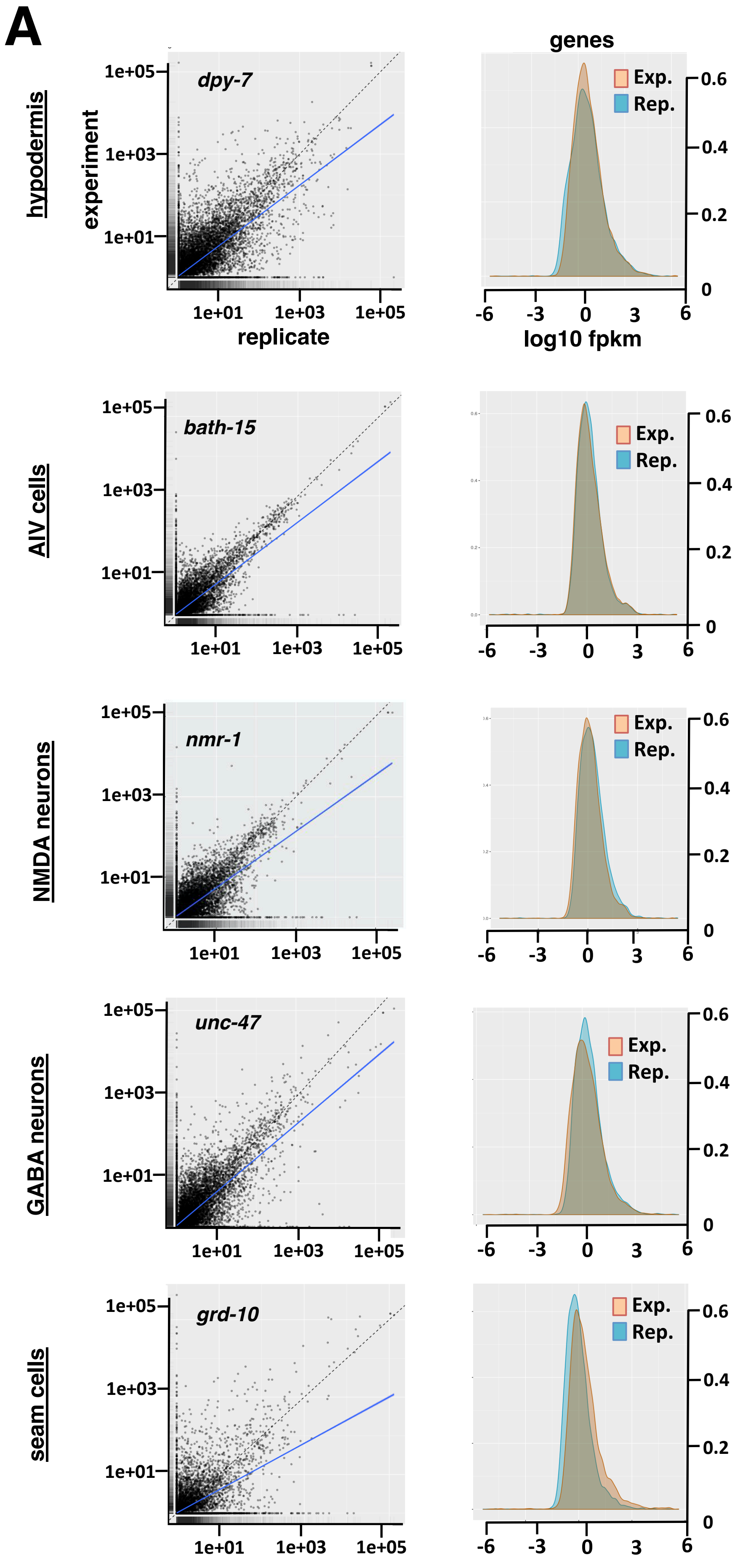

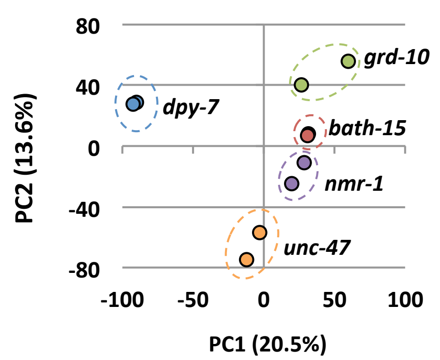

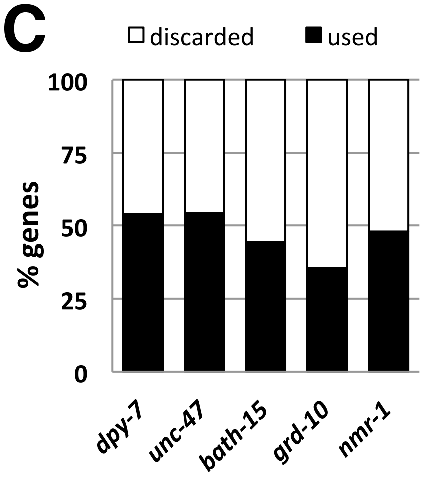

D

Extra chromosomal array transmission frequency

strain	Transmission (n)
Punc-47	87.2% (86)
Pgrd-10	100% (102)
Pdpy-7	92.4% (79)
Pbath-15	100% (77)
Pnmr-1	97.67% (172)

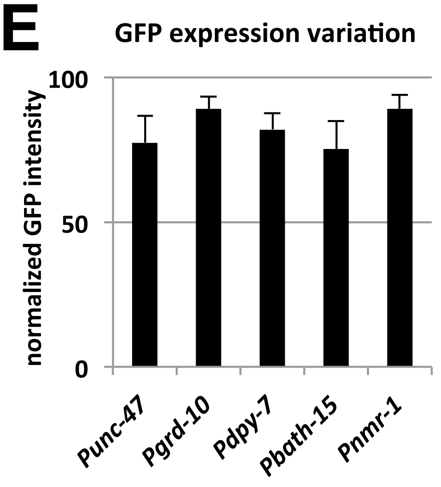

Figure S2

Figure S2: PAT-Seq sequencing results. A) Scatter plot of mapped genes from each tissue dataset displayed by fpkm value detected in each replicate on a logarithmic (log_{10}) scale to highlight similarity of detection between replicates. *dpy-7* (hypodermis), *bath-15* (AIV cells), *nmr-1* (NMDA neurons), *unc-47* (GABAergic neurons) and *grd-10* (seam cells). The right panels show the distribution of the fpkm values for all genes in control and replicate biological samples for each tissue. The plots were generated using the cummeRbund package v. 2.0. **B)** Principal Component Analysis (PCA) shows high correlation among duplicates within our datasets. **C)** In this study we have used only the top ~40-50% positive hits produced by Cufflinks. **D)** The transmission frequency of the extra chromosomal arrays in all strains is higher than 87%. **E)** Quantification of GFP expression using imageJ analysis on five different animals from each strain shows minimal variation across animals within each line.

Figure S3

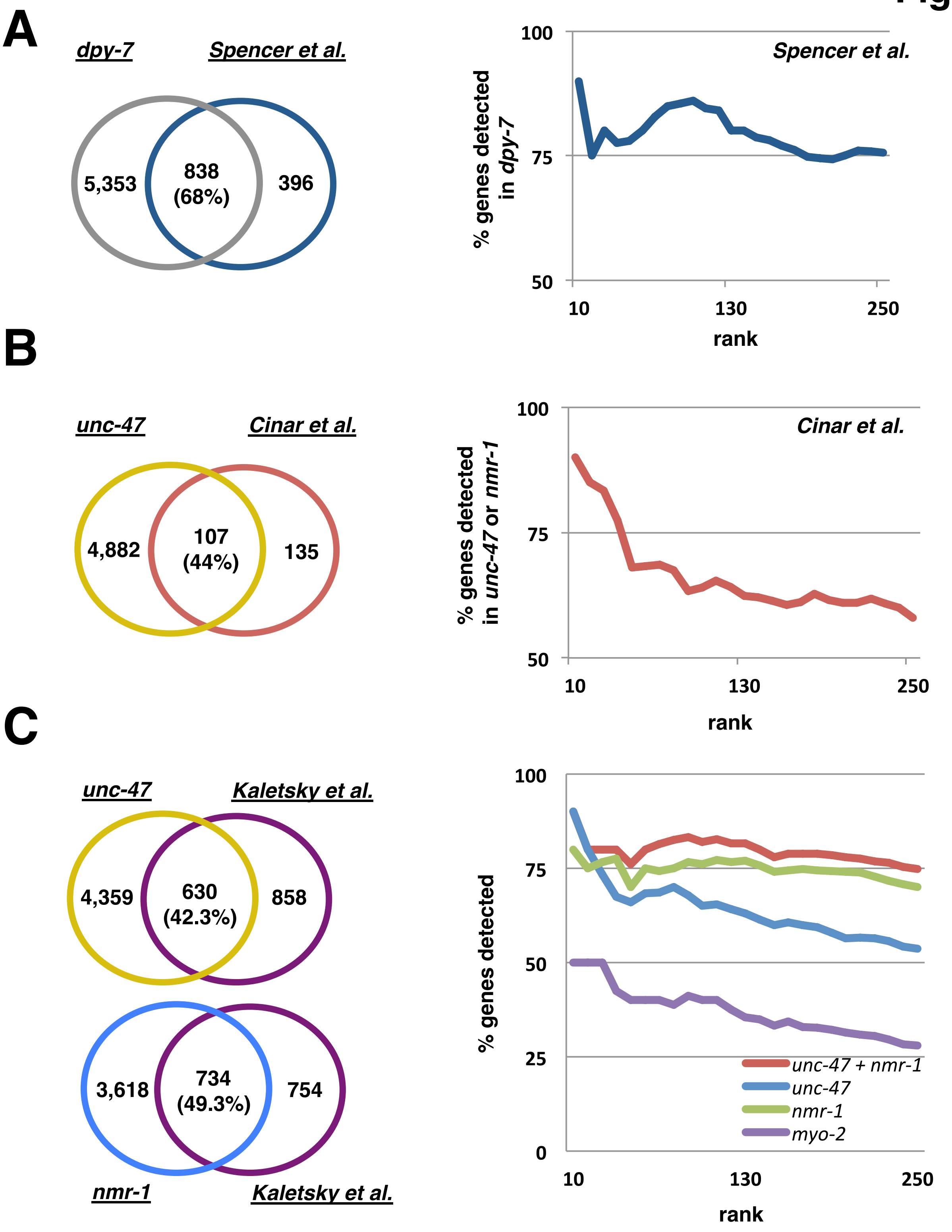

Figure S3: PAT-Seq comparison with other published mRNA-tagging derived datasets. We compared our PAT-Seq tissue transcriptomes with published tissue transcriptomes from **A)** hypodermis from Spencer et al. (n= 1,234). *Left:* Venn diagram showing 68% overlap with our hypodermis dataset. *Right:* Spencer et al. genes ordered by expression level (rank) detected in our dataset. We detect most of the top 100 expressed genes from the Spencer et al. dataset. **B)** GABAergic neurons from Cinar et al. (n= 242). **C)** Neuron datasets compared to Kaletsky et al. (n=1,488)

Figure S4

A

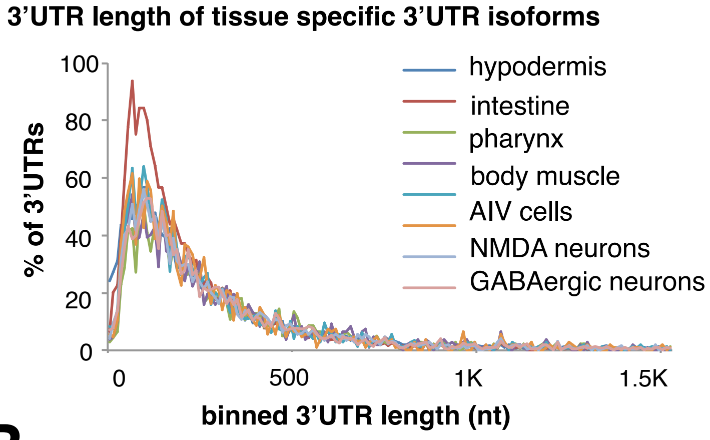

B

	tissue	median 3'UTR length (nt)
	**intestine (n= 8,004)**	**151**
	**hypodermis (n= 9,567)**	**176**
	**AIV cells (n= 2,163)**	**180**
	**seam cells (n= 1,576)**	**186**
	**NMDA neurons (n= 3,584)**	**194**
	**GABAergic neurons (n= 3,837)**	**196**
	**body muscle (n=1,500)**	**203**
	**pharynx (n= 1,869)**	**213**

C

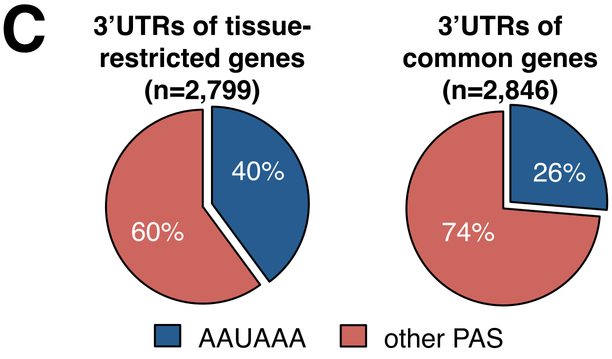

D

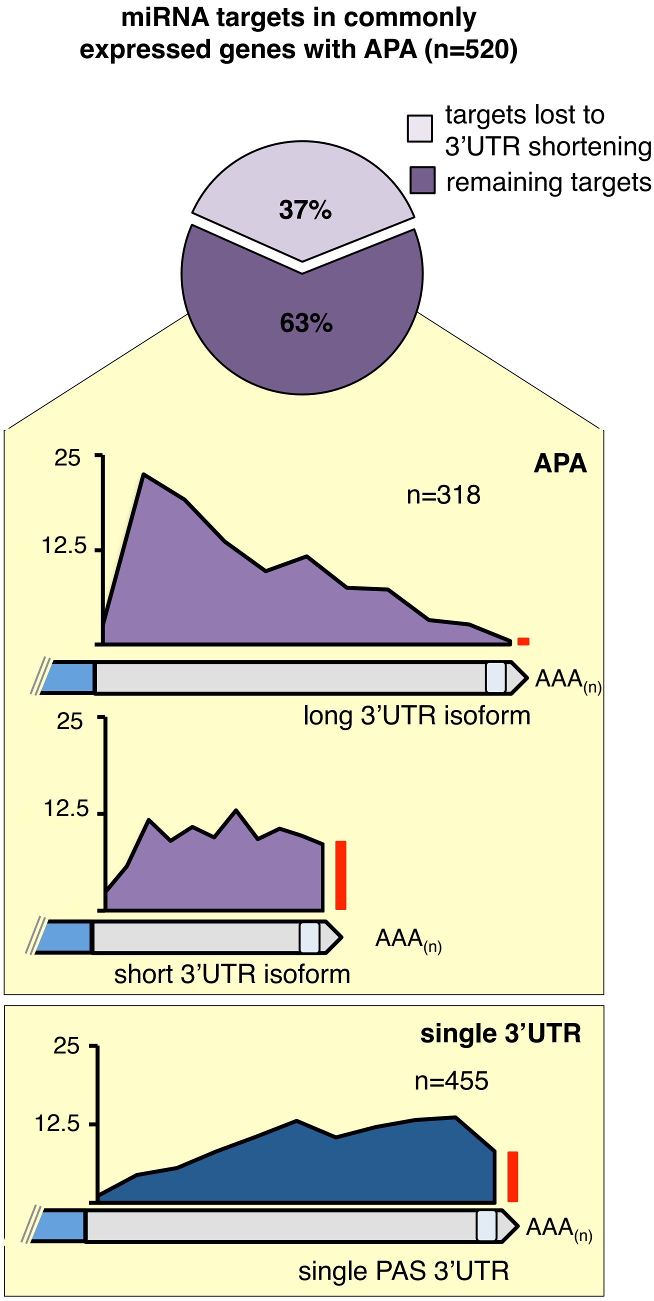

Figure S4: 3'UTR length in each tissue and PAS usage in tissue-restricted versus commonly transcribed genes. A) Histogram displaying the distribution of 3'UTR length in each tissue. The intestine expresses shorter 3'UTRs, on average. B) Median 3'UTR length for all genes expressed in each indicated tissue, sorted by ascending median length. Muscle tissue genes have a large median 3'UTR length. C) Pie charts displaying the proportion of 3'UTR isoforms having canonical PAS elements 'AAUAAA' or other PAS variants in tissue-restricted genes (left chart) or commonly transcribed genes (right chart). Commonly transcribed genes use non-canonical PAS elements more often than tissue-restricted genes. D) *Top*: Pie chart showing the portion of all miRNA targets in commonly transcribed genes that are lost due to expression of the short 3'UTR isoforms of each gene due to APA. Commonly transcribed genes lose ~43% of total miRNA targets due to tissue-specific APA. *Bottom*: We mapped the position of the 57% predicted miRNA targets in the longest and shortest 3'UTR isoforms (purple peaks), normalized by percent distance from the STOP codon. Many predicted miRNA targets overlap the PAS at the 3'end of the transcript in the short 3'UTR isoform compared with the long isoform (compare height of red bars). The same analysis performed on a dataset of the same number of 3'UTRs of genes that do not undergo APA (single PAS 3'UTR, blue peak) reveals an enrichment of miRNA targets near the PAS (red bar) that is more similar to the short 3'UTR isoforms of commonly transcribed genes.

Figure S5

predicted miRNA family targets enriched in each tissue transcriptome

miRNA	tissues: intestine	body muscle	pharynx	GABAergic neurons	NMDA neurons	seam cells	hypodermis	AIV cells
mir-2	1	1	1	1	1	1	1	1
mir-58	2	4	3	2	2	3	2	2
let-7	3	2	2	3	3	2	3	3
mir-44	4	3	4	4	4	6	4	4
mir-63	5	6	9	6	6	4	8	10
mir-50	6	7	6	7	8	10	7	5
mir-75	7	8	10	9	7	9	5	8
mir-1	8	12	11	5	11	7	10	7
mir-35	9	10	7	11	9	8	9	9
mir-51	10	5	5	8	5	5	6	6
mir-86	11	9	8	10	12	11	12	11
mir-72	12	13	13	13	13	13	13	13
mir-232	13	11	12	12	10	12	11	12
mir-87	14	15	15	14	16	16	14	15
mir-251	15	14	16	16	14	15	15	14
mir-49	16	16	14	15	15	17	16	16
mir-34	17	17	17	17	18	14	17	18
mir-46	18	18	18	18	17	18	18	17
lin-4	19	20	20	20	20	20	20	20
mir-231	20	19	19	19	19	19	19	19

predicted miRNA family targets lost to APA

miRNA	tissues: intestine	body muscle	pharynx	GABAergic neurons	NMDA neurons	seam cells	hypodermis	AIV cells
mir-2	18	13	7	13	13	6	6	11
mir-58	13	20	14	14	16	13	4	15
let-7	11	19	6	12	5	15	18	14
mir-44	12	9	4	9	6	8	17	8
mir-63	7	8	15	2	4	10	5	10
mir-50	2	6	10	7	2	3	19	7
mir-75	15	7	13	18	11	17	20	9
mir-1	19	11	11	17	19	9	9	17
mir-35	6	12	2	10	3	5	10	4
mir-51	20	15	8	19	10	16	15	5
mir-86	17	14	17	5	9	7	13	13
mir-72	4	16	1	6	1	2	8	1
mir-232	5	3	5	3	8	1	1	3
mir-87	9	2	3	1	7	4	3	6
mir-251	16	17	18	15	12	14	2	16
mir-49	1	1	12	11	17	18	12	18
mir-34	10	18	9	8	14	12	14	19
mir-46	8	10	16	4	20	11	11	12
lin-4	3	4	20	20	15	19	7	2
mir-231	14	5	19	16	18	20	16	20

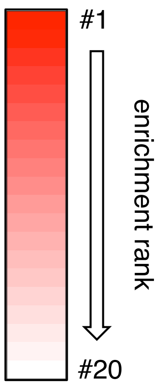

Figure S5: APA drives loss of distinct miRNA family targets. We ranked the overall abundance of PicTar and miRANDA predicted targets for each of the twenty *C. elegans* miRNA families (Alvarez et. al, 2010) in genes expressed in each tissue transcriptome (left) and those lost due to 3'UTR shortening through APA in each tissue (right). miRNA families that are lost to APA are largely different than those that are most enriched in each tissue transcriptome overall. Predicted targets of the *miR-72*, *mir-232* and *miR-87* miRNA families are frequently lost among tissues (dotted box).

Figure S6

A

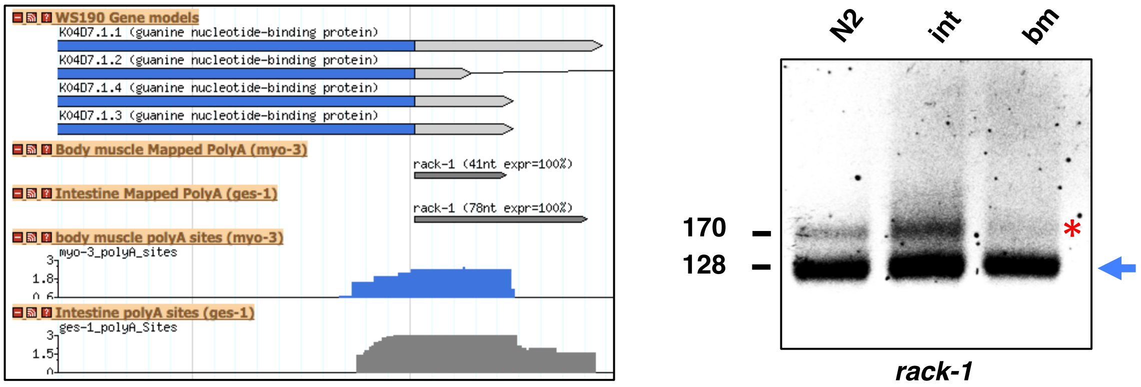

B

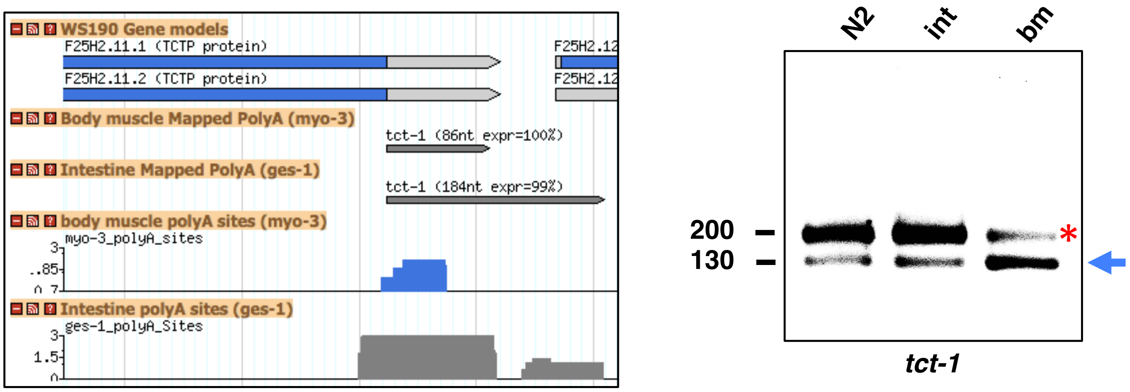

Figure S6: *rack-1* and *tct-1* are expressed with a short 3'UTR isoform in the body muscle and long 3'UTR isoform in the intestine. A) *Left*: diagram of clusters mapped for *rack-1* in body muscle (blue) and intestine (gray) tissues. *Right*: Results of 3'RACE experiments to amplify the 3'ends of *rack-1* in total RNA extracted from all tissues (N2), or mRNA samples prepared from intestine (int) or body muscle (bm) tissues using the PolyA-Pull immunoprecipitation (Blazie et. al, 2015). The long 3'UTR isoform of *rack-1* (red asterisk) was detected only in the intestine-specific mRNA prep, while only the short 3'UTR isoform (blue arrow) was detected in the body muscle. B) *Left*: diagram of clusters mapped for *tct-1* in body muscle (blue) and intestine (gray) tissues. *Right*: Results of 3'RACE experiments to amplify the 3'ends of *tct-1* in total RNA extracted from all tissues (N2), or mRNA samples prepared from intestine (int) or body muscle (bm) tissues using the PolyA-Pull immunoprecipitation (Blazie et. al, 2015). The long 3'UTR isoform of *tct-1* (red asterisk) is most abundant in the intestine mRNA prep, while only the short 3'UTR isoform (blue arrow) is instead most abundant in the body muscle.

Figure S7

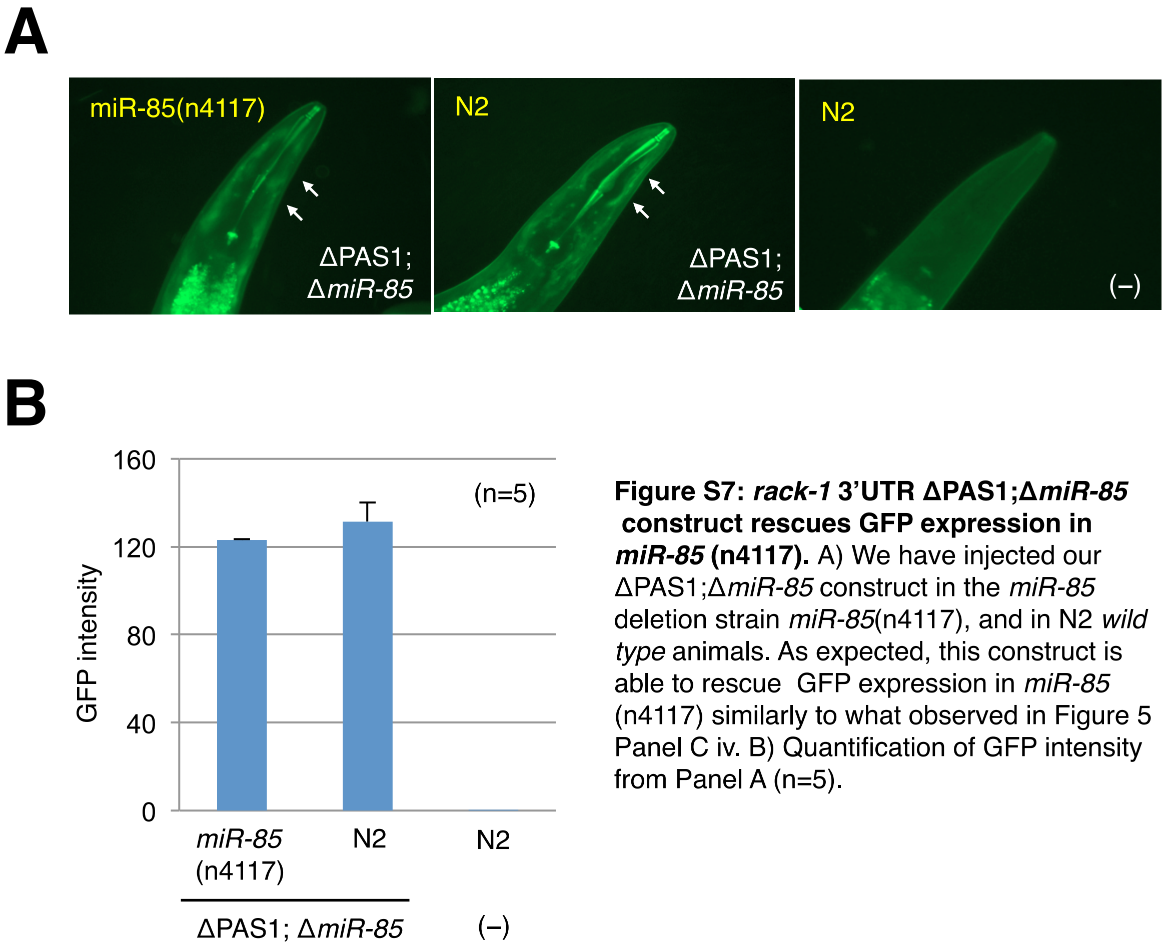

Figure S7: *rack-1* 3'UTR ΔPAS1;Δ*miR-85* construct rescues GFP expression in *miR-85* (n4117). A) We have injected our ΔPAS1;Δ*miR-85* construct in the *miR-85* deletion strain *miR-85*(n4117), and in N2 *wild type* animals. As expected, this construct is able to rescue GFP expression in *miR-85* (n4117) similarly to what observed in Figure 5 Panel C iv. B) Quantification of GFP intensity from Panel A (n=5).

Table S1

samples (tissue)			total reads	mapped (%)	not mapped	average depth
neurons	GABAergic neurons (*Punc-47*)	*experiment*	7,412,370	4,997,158 (67.42)	2,415,212	34.3x
		replicate	13,834,418	7,031,857 (50.83)	6,802,561	32.8x
	NMDA-type neurons (*Pnmr-1*)	*experiment*	7,048,055	5,171,858 (73.38)	1,876,197	30.1x
		replicate	6,526,023	4,241,115 (64.99)	2,284,908	21.9x
hypodermis	seam cells (*Pgrd-10*)	*experiment*	6,563,296	4,852,499 (73.93)	1,710,797	32.3x
		replicate	9,456,923	2,406,168 (25.44)	7,050,755	79.9x
	hypodermis (*Pdpy-7*)	*experiment*	31,509,607	10,288,238 (32.6)	21,221,369	44x
		replicate	14,090,409	6,222,500 (44.16)	7,867,909	31.4x
epithelial	AIV cells (*Pbath-15*)	*experiment*	6,297,159	4,591,797 (72.92)	1,705,362	31.5x
		replicate	8,151,065	5,850,351(71.77)	2,300,714	30.1x
muscle	pharynx (P*myo-2*)	*experiment*	31,370,767	27,174,301 (86.6)	4,196,466	12.5x
		replicate	29,596,195	23,954,109 (80.1)	5,642,086	25.9x
	body muscle (P*myo-3*)	*experiment*	30,993,699	24,557,134 (79.2)	6,436,565	12.6x
		replicate	33,770,647	27,760,544 (82.2)	6,010,103	9.9x
intestine	intestine (P*ges-1*)	*experiment*	36,296,454	25,478,178 (70.2)	10,818,276	60.5x
		replicate	29,897,356	20,870,860 (69.8)	9,026,496	46.9x

Table S1: Summary of results from PAT-Seq after deep sequencing.
Raw reads derived from tissue-specific mRNA libraries on the Illumina Hi-Seq Instrument, mapped to the *C. elegans* WS250 genome annotation.

Table S2

	samples (tissue)		genes	
**neurons**	GABAergic neurons (*Punc-47*)	*experiment*	4,399	3,011(*)
		replicate	4,608	
	NMDA-type neurons (*Pnmr-1*)	*experiment*	3,817	2,483(*)
		replicate	4,153	
**hypodermis**	seam cells (*Pgrd-10*)	*experiment*	3,051	1,208(*)
		replicate	1,972	
	Hypodermis (*Pdpy-7*)	*experiment*	5,393	3,786(*)
		replicate	5,596	
**epithelial**	AIV cells (*Pbath-15*)	*experiment*	3,539	2,112(*)
		replicate	3,397	
**muscle**	pharynx (*Pmyo-2*)	*experiment*	6,741	4,836(*)
		replicate	6,166	
	body muscle (*Pmyo-3*)	*experiment*	5,975	4,277(*)
		replicate	6,158	
**intestine**	intestine (*Pges-1*)	*experiment*	8,807	7,970(*)
		replicate	9,092	

Table S2: Summary of sequencing results after mapping genes to WS250.
Mapped reads from the tissue-specific mRNA libraries on the Illumina Hi-Seq Instrument. Genes and isoforms are mapped to the *C. elegans* WS250 genome annotation. Genes and isoforms marked with an asterisk correspond to genes and isoforms enriched in both biological duplicates (fpkm>=1).
